# MiR-25 Regulates Wwp2 and Fbxw7 and Promotes Reprogramming of Mouse Fibroblast Cells to iPSCs

**DOI:** 10.1371/journal.pone.0040938

**Published:** 2012-08-17

**Authors:** Dong Lu, Matthew P. A. Davis, Cei Abreu-Goodger, Wei Wang, Lia S. Campos, Julia Siede, Elena Vigorito, William C. Skarnes, Ian Dunham, Anton J. Enright, Pentao Liu

**Affiliations:** 1 Wellcome Trust Sanger Institute, Wellcome Trust Genome Campus, Hinxton, Cambridge, United Kingdom; 2 European Bioinformatics Institute, Wellcome Trust Genome Campus, Hinxton, Cambridge, United Kingdom; 3 Laboratory of Lymphocyte Signalling and Development, Babraham Institute, Cambridge, United Kingdom; Hemocentro de Ribeirão Preto, HC-FMRP-USP., Brazil

## Abstract

**Background:**

miRNAs are a class of small non-coding RNAs that regulate gene expression and have critical functions in various biological processes. Hundreds of miRNAs have been identified in mammalian genomes but only a small number of them have been functionally characterized. Recent studies also demonstrate that some miRNAs have important roles in reprogramming somatic cells to induced pluripotent stem cells (iPSCs).

**Methods:**

We screened 52 miRNAs cloned in a piggybac (PB) vector for their roles in reprogramming of mouse embryonic fibroblast cells to iPSCs. To identify targets of miRNAs, we made *Dgcr8*-deficient embryonic stem (ES) cells and introduced miRNA mimics to these cells, which lack miRNA biogenesis. The direct target genes of miRNA were identified through global gene expression analysis and target validation.

**Results and conclusion:**

We found that over-expressing miR-25 or introducing miR-25 mimics enhanced production of iPSCs. We identified a number of miR-25 candidate gene targets. Of particular interest were two ubiquitin ligases, Wwp2 and Fbxw7, which have been proposed to regulate Oct4, c-Myc and Klf5, respectively. Our findings thus highlight the complex interplay between miRNAs and transcription factors involved in reprogramming, stem cell self-renewal and maintenance of pluripotency.

## Introduction

Mouse and human fibroblast cells can be reprogrammed to iPSCs by expressing the four Yamanaka factors: Oct4, Sox2, Klf4 and c-Myc [1], [2], [3], [4], [5], [6], [7]. Other somatic cell types have also been reported to be reprogrammed to iPSCs [8], [9], [10], [11], [12]. Besides retroviral vectors, alternative routes are used to deliver the genetic factors such as lentivirus [2], adenovirus [13], plasmid-based vectors [14], episomal vectors [15], and the *piggyBac* (*PB*) DNA transposon [16], [17]. A growing number of genetic factors and chemical compounds are described to either improve reprogramming or even to replace one of the Yamanaka factors [18], [19], [20], [21], [22], [23], [24]. Moreover, perturbating several biological processes such as the cell cycle and DNA repair has profound effects on reprogarmming [25], [26], [27], [28].

MicroRNAs function to promote target mRNA degradation or as repressors of translation by binding the target sites usually located in the 3′ UTRs [29]. The long primary miRNAs (pri-miRNAs) are processed by the microprocessor complex, composed of the double-stranded RNA-binding protein DGCR8 and the RNase III enzyme DROSHA, into short hairpins called precursor miRNAs (pre-miRNAs) [30], [31], [32], [33]. These hairpins are exported to the cytoplasm and are processed by Dicer into mature miRNAs [34], [35]. Mouse ES cells express abundant miRNAs which play important roles in ES cell biology. As a consequence *Dicer*- or *Dgcr8*-deficient mouse ES cells are defective in cell cycle progression and differentiation [36], [37], [38]. These defects can be partially rescued by introduction of certain mature miRNAs. For instance, introduction of a subset of the miR-290 cluster is able to rescue the cell cycle defects in *Dgcr8-*deficient ES cells [39]. These miRNAs, which include miR-291-3p, miR-294 and miR-295, are thus named ES cell-specific cell cycle-regulating (ESCC) miRNAs based on their ability to regulate G1-S transition [39]. Moreover, proteins important in ES cell pluripotency are found to bind the promoter regions of miRNAs and regulate their transcription activities [40]. Therefore, miRNAs appear to have significant roles at the centre of the regulatory networks that control pluripotency. Indeed, overexpressing ESCC miRNAs can improve reprogramming by replacing *c-Myc* [41], and expressing the miR-302 cluster is sufficient to reprogram a cancer cell line to cells expressing pluripotency genes [42]. In a dramatic show of miRNA's function in reprogramming, Morrisey and colleagues recently demonstrated that expressing the miR-302/367 cluster alone is sufficient to reprogramme mouse and human fibroblast cells to iPSCs [43]. A combination of mir-200c plus mir- 302 and mir-369 family miRNAs is also shown to produce mouse and human iPSCs [44]. Conversely, inhibition of the let-7 family, which silences ES cell self-renewal by suppressing many of the same downstream targets that are indirectly activated by the ESCC miRNA family, promotes de-differentiation of somatic cells and reprogramming [45].

To identify additional miRNAs that affect reprogramming, we performed a genetic screen focusing on miRNAs that are expressed in mouse ES cells [46], [47], or over-expressed in cancer [48], [49]. From this screen, we found that overexpressing miR-25 substantially improved reprogramming, which was confirmed by introducing miR-25 mimics. The iPSCs produced were fully pluripotent as they contributed efficiently to both somatic cells and the germline in chimeras. Moreover, through bioinformatics analysis and experimental validation, we identified that miR-25 directly regulated Wwp2, an E3 ubiquitin ligase that targets Oct4 for ubiquitination, and Fbxw7, which is known to regulate c-Myc, Klf5 and other important factors. These results thus reveal a mechanism for miR-25 to regulate pluripotency genes and provide new information for efficient reprogramming.

## Results

### MicroRNAs that promote reprogramming

**Figure 1:**
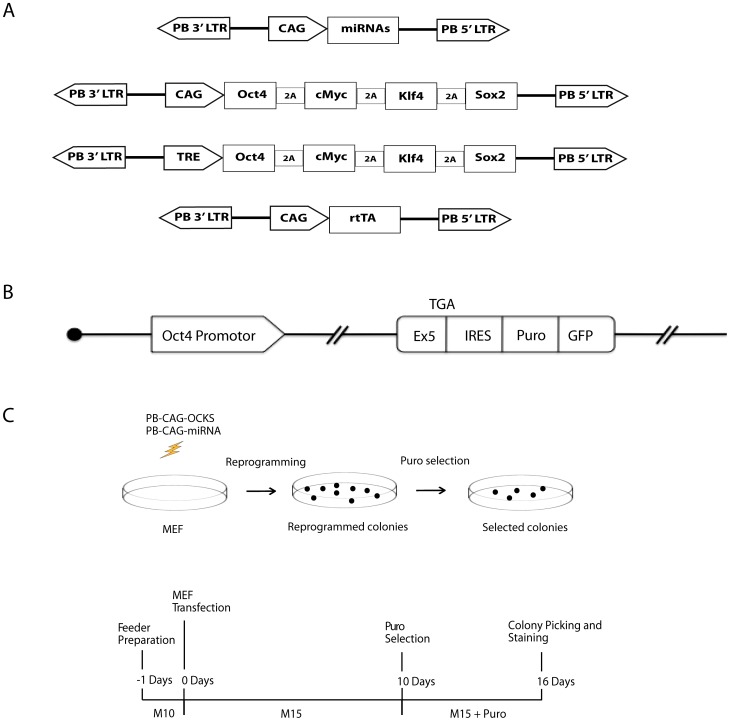
Reprogramming MEFs using PB transposons carrying the four Yamanaka factors and miRNAs. **a.** Schematic diagrams of the PB transposon constructs. Mouse cDNAs for *Oct4*, *c-Myc*, *Klf-4* and *Sox2* (OCKS) were cloned under the control of either the CAG promoter or the TRE (Tet responsive element). Expression of rtTA (reverse tetracycline transactivator) and the miRNAs was controlled by the CAG promoter. cDNAs of OCKS were joined by a linker encoding the 2A peptide. **b.** The *Oct4-*reporter allele. The *IRES-PuroGFP* cassette was targeted to the 3′ UTR of *Oct4* locus, immediately after the TGA stop codon in exon 5. **c.** Timeline of the reprogramming strategy. MEF cells were transfected with the transposon constructs and plated onto STO feeder cells. Puromycine (2.0 µg/ml) was added to select for *Oct4* expression on day 10. If the Dox-inducible transposon (PB-TRE-OCKS) was used, Dox (1.0 µg/ml) was added one day after transfection.

The *piggyBac* (*PB*) transposition works efficiently in mammalian cells. We have previously comprehensively characterized *PB* transposition in mammalian cells and used it in reprogramming experiments [16], [50], [51]. To start exploring the roles of miRNAs in reprogramming, we selected 52 miRNAs or miRNA clusters, based on their expression in ES cells or in tumour cells (Table S1) [46], [47], [48], [49]. These miRNAs were cloned in the PB vector by PCR-amplifying 500–800 bp genomic DNA fragments that encompass the ‘precursor’ miRNA sequences (Table S2). In these PB vectors, miRNA expression was under the control of the CAG promoter (PB-CAG-mir) (Fig. 1a).

ES cell pluripotency requires proper levels of *Oct4* (*Pou5f1*) expression [52], and activation of *Oct4* is a critical event in the reprogramming process [4]. We constructed and used an *Oct4*-reporter mouse line where an *IRES-PuroGFP* cassette was targeted to the 3′ UTR of the *Oct4* locus (Fig. 1b) [51]. Fully pluripotent iPSCs reprogrammed from these *Oct4*-reporter MEFs are resistant to 2.0 µg/ml puromycin (Puro) selection and identifiable as GFP^+^ in flow cytometry [51]. Therefore we use resistance to 2.0 µg/ml Puro or GFP expression to assess primary reprogrammed colonies, followed by comprehensive characterization.

To reprogram mouse embryonic fibroblasts (MEFs) to iPSCs, we constructed the PB transposon carrying the four Yamanaka factor cDNAs controlled either by the CAG promoter (PB-CAG-OCKS, for Oct4, cMyc, Klf4 and Sox2) for constitutive expression, or by the Tet-response element (PB-TRE-OCKS) for inducible expression by Doxycycline (Dox) (Fig. 1a). As the baseline reprogramming control, we transfected the PB-CAG-OCKS transposon to *Oct4*-reporter MEFs, and plated the cells on STO feeder cells (Fig. 1c). To examine the effects of miRNAs in addition to the Yamanaka factors on reprogramming, we co-transfected the PB-CAG-OCKS plasmid with each of the 52 PB-CAG-miRNA plasmids and selected the reprogrammed colonies for puromycin resistance (2.0 µg/ml). We usually began Puro selection 10 days after transfection when ES cell-like colonies started to appear. Puro^r^ colonies were subsequently picked for expansion and further analysis.

**Figure 2:**
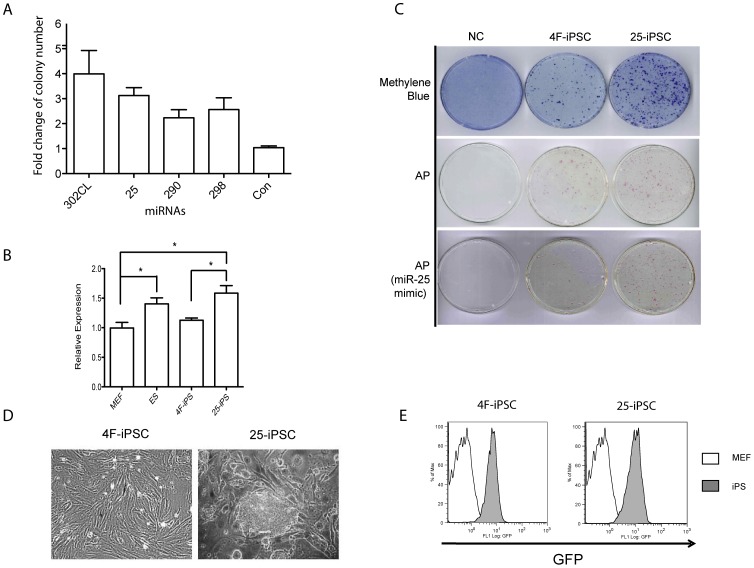
Co-expressing OCKS and microRNAs improved reprogramming. **a.** Four microRNAs: miR302 (cluster), miR-25/93, miR-290 and miR-298 were able to improve reprogramming. Shown here is the fold change of iPSC colony number (2.0 µg/ml Puro^r^). Fold changed was calculated based on the iPSC colony number using the four factors only (mean ± SEM. N = 4). **b.** qPCR analysis of the mature form of miR-25 expression. Expression was determined by qPCR and normalized using Sno202 miRNA. Data are mean ± SEM from three cell lines. * P<0.05. **c.** Reprogrammed colonies (2.0 µg/ml Puro^r^) stained either by Methylene blue or for Alkaline Phophatase activity (AP). NC: negative control using a PB-Neo plasmid with no reprogramming factors; 4F-iPSC: iPSCs reprogramming by expressing OCKS (Dox inducible); 25-iPSC: iPSCs reprogramming by expressing OCKS (Dox inducible) and miR-25. Mimic: Transfection used a miR-25 mimic instead of the PB-CAG-mir25. **d.** A primary iPSC colony reprogrammed with the four Yamanaka factors and miR-25. **e.** GFP expression from the *Oct4* locus in 4F-iPSCs and 25-iPSCs detected by flow cytometry.

Four out of the 52 tested miRNAs or miRNA clusters: the miR-302 cluster, miR-25, miR-290 and miR-298, gave substantially more Puro^r^ iPSC colonies (2–4 fold) than the control where only the four factors were used (PB-CAG-OCKS) (Fig. 2a). Identification of miR-302 and miR-290 in our screen confirmed the previous studies [41], [42]. However, we were unable to reproduce the result reported by Morrisey and colleagues [43] that expressing the miR-302/367 cluster alone is sufficent to reprogramme MEFs to iPSCs. This discrepancy is possibly caused by the miRNAs delivery approaches. We used the PB transposon where miR-302/367 cluster expression was controlled by the CAG promoter whereas in Morrisey's work, this cluster was delivered using a lentiviral vector. However, this possibility needs to be further investigated experimentally. Although miR-290 itself was not known to promote reprogramming, several members of the miR-290 family, miR-291-3p, miR-294 and miR-295, enhance reprogramming of MEFs in the absence of *c-Myc* [41]. The other two miRNAs identified in this screen, miR-25 and miR-298, were not previously reported for their role in ES cells or in reprogramming when the project started. We therefore chose initially to further characterize miR-25 since it was found to be expressed in ES cells (Fig. 2b), and its mature sequence is conserved across all vertebrate genomes examined (Table S3). On the other hand, miR-298 expression was not found in ES cells [47].

To confirm the effects of miR-25 over-expression on reprogramming, we transfected 1×10^6^ MEFs with PB-TRE-OCKS, PB-CAG-rtTA and PB-CAG-mir25 (Fig. 1a). Dox was added to the culture media to induce expression of the four Yamanaka factors. Again expression of miR-25 substantially increased the number of Puro^r^ iPSCs (Fig. 2c–d). The iPSC colonies were picked 16 days after transfection, and initially expanded in M15 plus LIF media. Dox was subsequently removed from the culture during colony expansion and the medium was changed from M15 plus LIF to the 2i medium. This medium is considered to be able to select and maintain ground state or naïve pluripotent ES cells by inhibiting the activities of ERK and GSK3β [53]. iPSCs produced from using either the 4 Yamanaka factors (4F-iPSC) or 4F plus miR-25 (25-iPSC) proliferated well in the 2i medium. Consistent with their resistance to 2.0 µg/ml Puro, the cells also expressed GFP in flow cytometry thus demonstrating robust expression of the endogenous *Oct4* locus (Fig. 2e).

The miR-25 genomic DNA cloned in the PB also contains miR-93 (Table S2). However, we did not detect noticeable effect on reprogramming by expressing miR-93 from another genomic DNA containing only miR-93 (Table S2). To further examine the specific effect of miR-25 on reprogramming, we repeated the above reprogramming experiments using a miR-25 mimic instead of the genomic DNA containing miR-25. Adding miR-25 mimic increased AP^+^ colony number similar to using PB-CAG-mir-25/93 (Fig. 2C)

### Characterization of iPSCs produced by over-expressing miR-25

**Figure 3:**
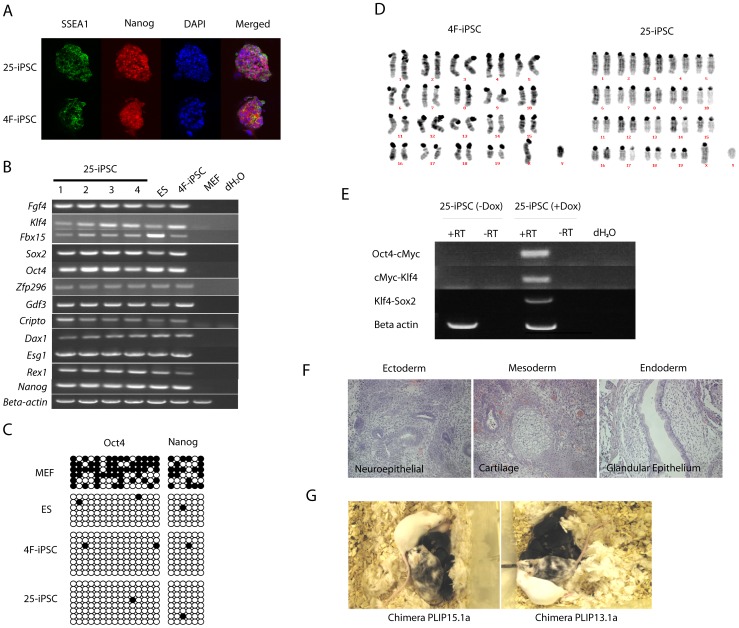
Characterization of 25-iPSCs. **a.** Immunofluorescence-staining of iPSCs to detect SSEA1 and Nanog expression. DNA was stained with DAPI (blue). **b.** RT-PCR analysis of pluripotency gene expression in 25-iPSCs and 4F-iPSCs. MEFs and ES cells serve as the negative and positive controls, respectively. Beta-actin was used as the PCR control. dH2O: no DNA template. **c.** Bisulfide genomic sequencing of the promoter regions of *Oct4* and *Nanog* loci to detect CpG methylation. Open and filled circles represent unmethylated or methylated CpG dinucleotides, respectively. **d.** Normal karyotypes in a male 4F-iPS cell and a male 25-iPS cell. **e.** No exogenous Yamanaka factor expression in 25-iPSCs. RT-PCR was performed using primers to amply OCKS junctions (Primer sequences are in Table S2). Robust expression from PB-TRE-OCKS was detected in the presence of Dox. No expression was found if Dox was removed from the media. +RT: reverse transcriptase; -RT: no reverse transcriptase in reverse transcription. Beta-actin was used as the PCR control. **f.** Haematoxylin eosin stained sections of teratomas derived from 25-iPSCs. Cells representing all three germ layers were readily identifiable. **g.** Germline-transmission pups (agouti) from two chimeras derived from 25-iPSCs that were crossed to wild-type C57BL6 (albino) females.

Dox induced iPSCs of both 4F-iPSC and 25-iPSC were expanded for over 20 passages in the 2i medium without Dox. Both iPSCs expressed pluripotency markers as well as a panel of pluripotency-associated genes at levels comparable with that in ES cells (Fig. 3a and 3b). However, compared to MEFs and 4F-iPSCs, 25-iPSCs expressed higher levels of miR-25 (Fig. 2b). Bisulphite genomic DNA sequencing analysis of the promoter regions of *Oct4* and *Nanog* loci revealed extensive demethylation in both 25-iPS and 4F-iPS cells as seen in ES cells, thus further confirming activation of these pluripotency gene loci (Fig. 3c). Importantly, even after extensive *in vitro* passage, these iPSCs retained a normal karyotype (Fig. 3d). To ensure that the exogenous factors were not expressed in the absence of Dox, we designed primers to amplify junction fragments between the Yamanaka factor cDNAs in PB-TRE-OCKS, and performed RT-PCR using RNA samples from iPSCs growing in the presence or the absence of Dox. As shown in Fig. 3e, Dox induced robust expression of the Yamanaka factors, while withdrawing Dox completely shut down their expression in the majority of examined iPSC lines (Fig. 3e).

To determine differentiation potentials of iPSCs, we injected 4F- and 25-iPSCs into F1 mice of C57BL/6 and 129S5 because *Oct4*-reporter MEFs had a C57BL/6/129S5 mixed genetic background. Both 4F- and 25-iPSCs efficiently formed teratomas containing tissues of all three germ layers (Fig. 3f). The 4F- and 25-iPSCs were also injected to C57BL/6 (albino) blastocysts for chimera production. Chimeras with extensive donor cell contribution were obtained from both iPSCs (Fig. 3g). Two male chimeras produced from injecting 25-iPSCs were crossed to wild type C57BL/6 (albino) females to test germline contribution of the iPSCs in the chimeras. In the first eight litters, 50 out of 52 pups were agouti coat colour indicative of very efficient contribution of 25-iPSCs in the germline of the chimeras (Fig. 3g). Similarly, two male chimeras produced from 4F iPSCs were also crossed to wild type C57B6 females (albino). Out of total 32 pups in the first four litters, 24 were agouti. Since the Dox induced iPSCs did not express detectable levels of exogenous factors in the absence of Dox (Fig. 3E), chimeras derived from either 4F- or 25-iPSCs did not develop any tumour or abnormalities in the aging period (14 months, n = 10).

**Figure 4:**
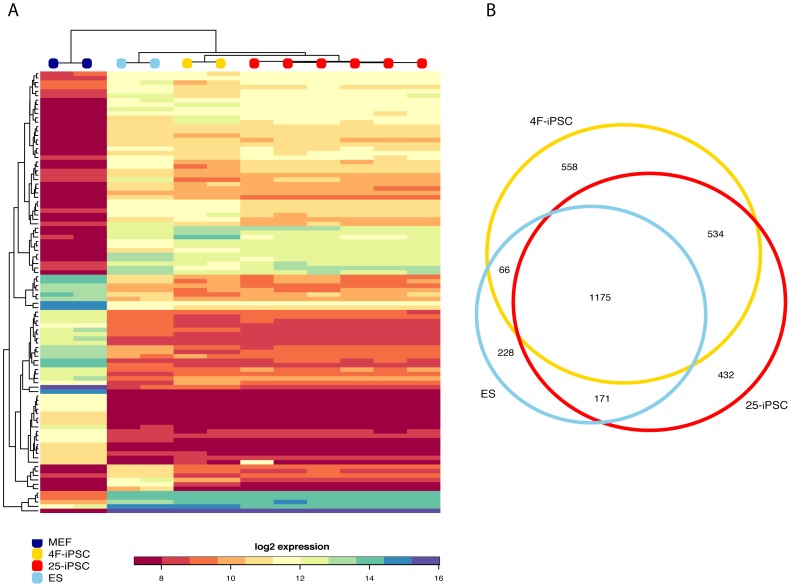
Genome-wide gene expression analysis in iPSCs and ES cells. **a.** Heatmap showing the (log2) absolute expression intensities in MEFs, 25-iPS, 4F-iPS and ES cells of the top 100 differentially expressed genes between MEFs and ES cells. Expression profiles are clustered based on correlation, and the dendrogram represents the relationship between different cell types. **b.** Venn diagram showing the number of shared differentially expressed genes among 25-iPSCs, 4F-iPSCs and ES cells when compared to MEFs.

To further characterize the 4F- and 25-iPSCs, we performed genome-wide gene expression microarray analysis. Expression profiles of 4F-iPSCs and 25-iPSCs were highly correlated, and clustered together with the profiles of ES cells and distinct from those of MEFs (Fig. 4a and b).

### Identification of miR-25 targets

Dgcr8 is a critical component of the complex that processes primary miRNAs. *Dgcr8*-deficient ES cells have defects in miRNA biogenesis [38]. These cells thus provide an ideal platform to facilitate identifying potential miRNA targets once a mature miRNA is introduced into these ES cells. The obvious advantage of this system over transfecting miRNA mimics in another cell type (e.g. HeLa or normal ES cells) is that the target sites are not saturated by the endogenous miRNAs and there will be no functionally redundant miRNAs to confound the results.

**Figure 5:**
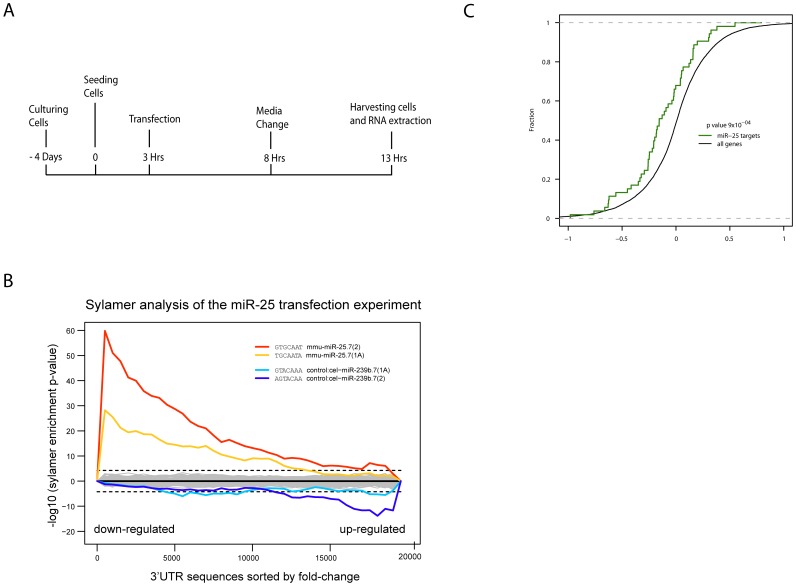
Identification of miR-25 targets. **a.** Timeline of miR-25 mimic transfection into the *Dgcr8*-deficient ES cells for discovering transcripts that were repressed by miR-25. The *C. elegans* miRNA, cel-miR-239b, was used as the negative control. **b.** Sylamer plots following the analysis of microarray data. The x-axis represents the 3′UTR sequences of all transcripts, sorted from the most down-regulated after miR-25 transfection compared to the cel-miR-239b transfection. Sylamer was used to analyze the enrichment or depletion of all possible heptamers matching mouse miRNA seeds, at every 500 sequences of the gene list. The horizontal dashed lines denote Bonferroni corrected p-value thresholds of 0.05. Words matching the mmu-miR-25 and cel-miR-293b seeds are highlighted. **c.** Cumulative fraction plot of the fold-changes obtained by comparing 25-iPS *vs* 4F-iPS microarrays. The fold-changes for all genes are indicated by the black curve; the green curve represents the 54 miR-25 targets from Table S4.

We thus used a *Dgcr8*-deficient ES cell line where one allele was mutated by gene trapping and the other allele contained a targeted gene trap cassette (Davis et al. co-submission with this manuscript). To identify the direct target genes of miR-25, we introduced either a mimic version of miR-25 or a control miRNA (C. elegans miRNA, cel-miR-239b) (Fig. 5a) into the *Dgcr8*-deficient ES cells and generated gene expression profiles using Illumina BeadChip microarrays. Potential miR-25 target genes were expected to be down-regulated in cells with the miR-25 mimic but not in control cells. We used Sylamer to further characterise the effect of miR-25 expression [54]. Briefly, Sylamer tests for miRNA effects by searching sorted lists of genes for enrichment or depletion of words complementary to all possible miRNA “seed” regions, which are the most important determinants of target specificity. The 3′UTR sequences of all the transcripts profiled on the microarrays were sorted starting with the most down-regulated in the miR-25 transfection experiment. The only 7mers that were enriched within the 3′UTRs of these down-regulated genes correspond to words complementary to the miR-25 seed sequence, indicating that the experimental profile can be used to derive lists of genes enriched for direct targets of miR-25 (Fig. 5b). From this experiment we obtained a list of 72 transcripts, which were down-regulated after transfection of the miR-25 mimic, with a fold change of at least 1.2 and with an estimated false discovery rate less than 10%. Of these genes, 54 possessed at least one seed-matching site for miR-25 in their 3′UTR and constitute the initial set of experimentally derived miR-25 targets (Table S4). To test if these targets are being actively repressed by miR-25 in our iPSCs, we compared the 25-iPS microarray samples against the 4F-iPS samples. We observed that these miR-25 candidate targets were down-regulated in the 25-iPS cells (*p* value 9×10^−4^ by a Wilcoxon signed-rank test) (Fig. 5*C*). From these targets, we prioritized a list of 12 gene by selecting those that were also up-regulated in the *Dgcr8*-deficient cells, besides being down-regulated in 25-iPSCs relative to 4F-iPSCs (Table 1). Of particular interest on this gene list were *Wwp2* and *Fbxw7*, which have been shown to modulate *Oct4* and *c-Myc* respectively [55], [56], [57], [58], [59], [60].

**Table 1:** Candidate target genes of miR-25.

Gene Symbol	Description	miR-25 sites^a^	miR25 transfection^b^		Dgcr8 KO^c^		25-iPS vs 4F-iPS^d^	
			FC^e^	Adj. P. val^f^	FC^e^	Adj. P. val^f^	FC^e^	Adj. P. val^f^
**Acbd4**	acyl-Coenzyme A binding domain containing 4	1	−1.3	6E-02	1.4	4E-05	−1.2	2E-05
**Adam23**	a disintegrin and metallopeptidase domain 23	3	−1.6	1E-02	2.2	6E-08	−1.2	4E-03
**Fbxw7**	F-box and WD-40 domain protein 7	2	−1.6	8E-03	1.3	2E-02	−1.7	2E-09
**Lmbr1l**	limb region 1 like	1	−1.6	7E-03	1.2	4E-03	−1.3	5E-05
**Nck2**	non-catalytic region of tyrosine kinase adaptor protein 2	1	−1.6	9E-02	1.2	3E-03	−1.1	5E-02
**Plekhm1**	pleckstrin homology domain containing, family M (with RUN domain) member 1	2	−2.0	2E-02	1.9	2E-07	−1.2	5E-03
**Rab8b**	RAB8B, member RAS oncogene family	1	−1.8	6E-03	1.5	2E-07	−1.2	1E-02
**Tmem184b**	transmembrane protein 184b	1	−1.3	5E-02	2.5	6E-09	−1.1	9E-02
**Ttc39b**	tetratricopeptide repeat domain 39B	3	−1.6	7E-02	1.4	2E-02	−1.2	3E-04
**Whsc1l1**	Wolf-Hirschhorn syndrome candidate 1-like 1	2	−1.4	3E-02	1.5	9E-06	−1.3	3E-06
**Wwp2**	WW domain containing E3 ubiquitin protein ligase 2	1	−1.5	8E-02	1.2	3E-03	−1.1	9E-04
**zinc and ring finger 2**	zinc and ring finger 2	1	−1.8	3E-02	1.2	1E-02	−1.1	5E-02

a number of sites in the 3′UTR that are complementary to a 7 nt miR-25 seed region (GTGCAAT or TGCAATA).

b miR-25 transfection in Dgcr8 KO cells compared to a control transfection.

c Dgcr8 KO cells compared to Dgcr8 Het cells.

d 25-iPS cells compared to 4F-iPS cells.

e expression fold change.

f *p* values adjusted for multiple testing (Benjamini & Hochberg).

### Validation of wwp2 and fbxw7 as miR-25 targets

**Figure 6:**
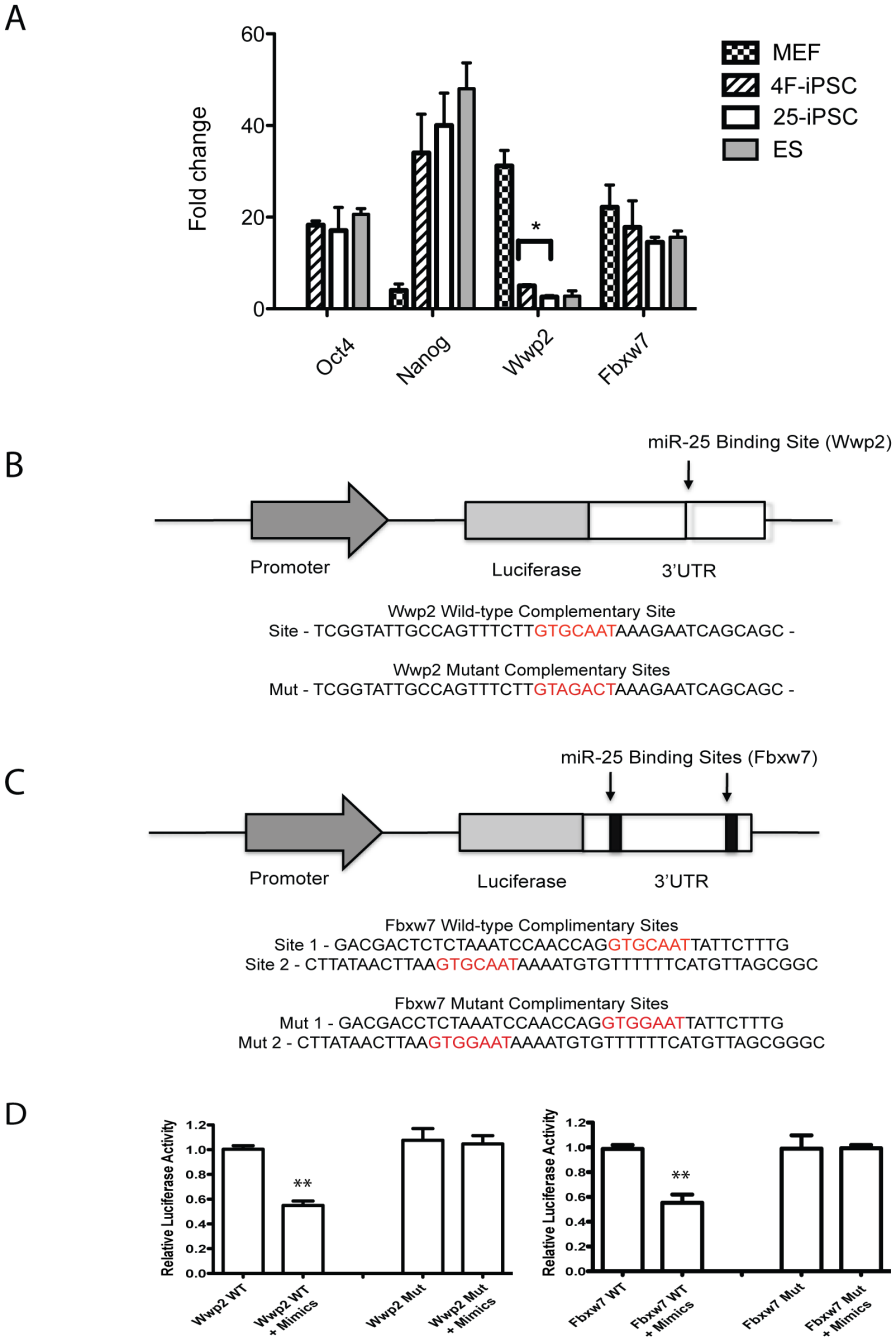
Validation of miR-25 targets. **a.** Realtime RT-PCR analysis of expression of *Oct4*, *Nanog*, *Wwp2* and *Fbxw7* in MEFs, iPSCs and ES cells. A significant decrease of *Wwp2* was detected in 25-iPSCs, compared to 4F-iPSCs. Gene expression was normalized against *Gadph*. Error bar (mean ± SEM. N = 3 cell lines). * P<0.05 compared with 25-iPS cells for Wwp2 expression. **b–c.** Luciferase reporter assay to the validate miR25 binding sites in the 3′UTRs of *Wwp2* and *Fbxw7*. Schematic diagrams of the reporters for *Wwp2* (**b**) and *Fbxw7* (**c**). The wild type and mutated miR-25 binding sites (highlighted in red) were indicated. The 3′ UTR of *Wwp2* has one miR-25 binding site, whereas *Fbxw7* has two. The 3′UTR genomic regions amplified for both *Wwp2* and *Fbxw7* are about 1.6 kb. **d.** Luciferase reporter assays for validating *Wwp2* and *Fbxw7* as direct targets of miR-25. Wild-type (Wt) or Mutant (Mut) reporter plasmids were co-transfected with miR-25 mimic or a control mimic into HeLa S3 cells to assay for the miR-25 dependent repression of *Wwp2* and *Fbxw7* reporter. Data are presented as mean ±SEM (N = 3). Comparison between groups were performed using one-way ANOVA followed by the Newman-Keuls post test to determine statistical significance. ** P<0.001.

We next proceeded to validate the experimentally predicted miR-25 targets. We first used quantitative (real-time) RT-PCR (qRT-PCR) to examine the expression of two selected miR-25 targets, *Wwp2* and *Fbxw7*, as well as the pluripotency markers *Oct4* and *Nanog* in MEF, 4F-iPSCs, 25-iPSCs and mouse ES cells. No significant difference in the expression of *Oct4* or *Nanog* was detected, possibly due to the tightly regulated expression of pluripotency genes in ES cells and in iPSCs. Wwp2 is expressed at much higher levels in MEFs than in ES cells (Fig. 6a). The level of the *Wwp2* transcript was significantly lower in 25-iPSCs compared to 4F-iPSCs, and was comparable to that in ES cells (Fig. 6a). Although we consistently detected lower *Fbxw7* expression in 25-iPSCs and ES cells than in 4F-iPSCs, this decrease was not statistically significant (Fig. 6a). The expression results were thus consistent with the predictions of *Wwp2*, and to a certain degree *Fbxw7*, as the potential targets of miR-25.

To demonstrate that miR-25 directly regulates *Wwp2* and *Fbxw7*, we performed the luciferase reporter assay based on pmirGLO Dual-Luciferase system where the full-length 3′ UTR of either *Wwp2* or *Fbxw7* was inserted into the 3′ side of the firefly luciferase gene (luc2) (Fig. 6b–c).

The wild type 3′ UTR of *Wwp2* carries one miR-25 target site. We introduced a point mutation into this target site, which would abolish miR-25 binding based on computational prediction. The reporter plasmids carrying either the wild type or the mutant 3′ UTR of *Wwp2* were co-transfected with the miR-25 mimics or the negative control mimics into HeLa S3 cells. The negative control mimics did not have any significant effect on reporter activity. However, the miR-25 mimic significantly repressed luciferase activity from the wild type reporter plasmid (Fig. 6d). Once the miR-25 binding target site was mutated, the repressing effect of the miR-25 mimic was lost (Fig. 6d). These results thus confirmed the critical role of the miR-25 binding site in regulating the luciferase activity in the reporter plasmid and thus *Wwp2*.

The 3′ UTR of *Fbxw7* has two miR-25 binding sites. Point mutations were subsequently introduced into both sites in the mutant reporter plasmid. Again, we co-transfected the reporter plasmids with the miR-25 mimics and the negative control mimic into HeLa S3 cells. The negative control mimic did not show any effect on the reporter activity, whereas the miRNA-25 mimics caused significant repression on the luciferase activity from the wild type reporter but not the mutated reporter (Fig. 6d). These data thus confirmed *Fbxw7* as another direct target of miR-25.

## Discussion

In an effort to identify new genetic factors in reprogramming, we screened 52 miRNAs/clusters that are expressed in ES cells or over-expressed in cancer. Two candidates, miR-25 and miR-298, were found to substantially improve reprogramming with the four Yamanaka factors. We focused on characterizing miR-25 in this study as miR-25 had a strong phenotype in promoting reprogramming, is highly conserved during evolution and is expressed in ES cells. The iPSCs produced by overexpressing miR-25 together with the four Yamanaka factors were pluripotent stem cells, based on their gene expression profiles, and on their ability to contribute to both the somatic lineages and the germline in chimeras. Further experiments designed to elucidate the contribution of miR-25 to this process combining computational prediction and experimental analysis using *Dgcr8*-deficient ES cells, demonstrated that miR-25 regulates a number of genes in mouse ES cells. We chose *Wwp2* and *Fbxw7* for further analysis and confirmed that miR-25 directly regulated *Wwp2* and *Fbxw7* in the luciferase reporter assay.

Wwp2, an E3 ubiquitin ligase, promotes Oct4 degradation by ubiquitination in both mouse and human ES cells [55], [56], thus suggesting a potential mechanism for improving reprogramming efficiency by miR-25. Importantly, Wwp2 expression levels in 25-iPSCs were slightly but consistently lower than that in iPSCs reprogrammed by the four Yamanaka factors alone (Fig. 6a). We did not however detect an obvious increase of Oct4 protein levels in 25-iPSCs (data not shown). The stable Oct4 expression level is likely caused by the pluripotent gene expression regulatory circuit in iPSCs where Oct4 is regulated by multiple factors. Fbxw7, on the other hand, is a component of SCF ubiquitin ligases that catalyzes the ubiquitination of cyclin E, Notch, c-Jun and c-Myc [57], [58], [59], [60]. Interestingly, Fbxw7 also directly targets Klf5 for ubiquitination and degradation [61]. Klf2, Klf4, and Klf5 are the core KLF protein circuitry with redundant function in regulating self-renewal of ES cells and Nanog expression [62], and Klf2 and Klf5 can replace Klf4 in reprogramming [63]. Thus, repression of Fbxw7 induced by over-expression of miR-25 might also contribute to improving reprogramming through upregulation of Klf5. While this manuscript was under preparation, miR-25 was shown to directly regulate p53 in tumour cells, and possibly p21 and Tgfβ signalling in MEFs [64], [65]. Both p53 and Tgfβαρε implicated in reprogramming [66], [67]. MiR-25 has also been recently suggested to function in IGF signalling, Wnt signalling and apoptosis in several cell types [68], [69], [70], [71]. In this study, we identified a different set of miR-25 targets in Dgcr8-deficient mouse ES cells. These targets include Wwp2 and Fbxw7, which may regulate three of the four reprogramming factors, Oct4, cMyc and Klf5. Other miR-25 targets identified in this study still remain to be characterised for their role in reprogramming. Our results, together with the other studies, show that, miR-25 promotes reprogramming by several mechanisms and possibly at distinct reprogramming stages. Given the conservation of miR-25 mature sequence during evolution and its role in regulating pluripotency genes, it will be interesting to determine whether its role is conserved in reprogramming human cells.

## Materials and Methods

### Ethics statement

All animal experiments were performed in accordance with the UK's 1986 Animals Scientific Procedure Act and local institute ethics committee regulations. Wellcome Trust Sanger Institute Ethical Review Committee approved this study.

### Construction of transposon plasmids encoding microRNA and the 4 factors

Candidate miRNAs were selected from published papers based on their expression in mouse ES cells, in the mouse oocyte or in cancer [40], [42], [46], [47], [48], [49], [77], [78], [79], [80], [81], [82], [83], [84] (Table S1). Genomic DNA fragments harbouring miRNAs were cloned into a PB vector carrying a CAG promoter-driven expression cassette (PB-CAGG-miRNA). cDNAs of Oct4, Sox2, Klf4 and c-Myc were cloned into a PiggyBac vector under either the control of the CAG promoter or the Tetracycline operator (PB-CAG-OCKS and PB-TRE-OCKS). The cDNAs of the 4 factors were generated by PCR amplification to insert 2A peptide sequence in between the four cDNAs. A separate PB vector, with M2rtTA insert was also cloned under the tetracycline operator (PB-TRE-rtTA). All plasmids had the pBlueScript backbone.

### Reprogramming of MEFs and iPS cell culture

MEFs with Oct4-GFP-Puro reporter allele were prepared from 13.5 d.p.c. embryos (C57BL/6 and 129S5 F1) cultured in M10 media: DMEM containing 10% FBS (HyClone), 1,000 units/ml Penicillin, 1,000 ug/ml Streptomycin, 2.92 mg/ml L-Glutamine and 1× non essential amino acids (Invitrogen). Individual PB-miRNAs were co-transfected with PB-OCKS into MEFs. Electroporation was performed using an Amaxa device to introduce 2 µg of PB-CAG-OCKS transposon and 2 µg of PB-CAG-miRNA transposon. The cells were then allowed to be reprogrammed to iPSCs in normal ES cell M15 media: DMEM containing 15% of FBS (Hyclone), 1,000 units/ml Penicillin, 1,000 ug/ml Streptomycin, 2.92 mg/ml L-Glutamine, 1× non essential amino acids, 0.1 mM 2-mercaptoethanol and 1,000 Uml^−1^ Leukemia inhibitory factor (LIF, Chemicon). For the Tet-inducible system, a PB-Tet-OCKS was co-electroporated with 1.0 µg of PB-CAG-rtTA plasmid. After 10 days of Doxycyclin induction, Puromycin selection (2.0 µg/ml) was applied. The plates were stained with methyl Blue or for Alkaline Phosphatase activities to score iPSC colonies. The iPSC colonies were cultured for 4 days in 2i media (iSTEM, Stem cell sciences). All ES cells and iPSCs were cultured on Mitomycin C–treated STO feeder cells.

### Transfection of the miR-25 mimics in reprogramming

MEF cells were transfected with 2.0 µg of PB-CAG-OCKS plasmid, together with 16 nM of miRNA-25 mimic (Dharmacon) using the Amaxa transfection device. At 3, 6 and 9 days post initial transfection, these cells were transiently transfected with 16 nM of miRNA-25 mimic (Dharmacon) with Lipofectamin 2000 (Invitrogen) according to manufacture's instructions. Reprogrammed colonies were stained with Alkaline Phosphate.

*Dgcr8*-deficient ES cell lines were maintained in GMEM media supplemented with 100 µg/ml hygromycin B (Calbiochem). These cells were split every 2–3 days as they approached confluency.

### Flow cytometry

Flow cytometric analysis was performed using iPSCs growing in 96-well plates to detect the GFP expression under the *Oct4* promoter. We used a Cytomics FC500 series (Beckman Coulter) in this work.

### RT-PCR

Reverse transcription reactions were performed using SuperScript II (Invitrogen) and the Oligo dT_20_ primer. PCR was then carried out using the primers for ES cell marker genes listed in table 1 and an Extensor Hi-Fidelity PCR master mix 2 (ABgene). PCR was performed using the following settings: 94°C for 4 min, followed by 30 cycles of 94°C for 30 sec, 65°C for 30 sec and 68°C for 45 sec. Final incubation was at 68°C for 10 min.

### Real time PCR

Quantitative real time PCR was performed using Taqman Gene Expression assays (Applied Biosystems) according to manufacture's instructions. The amount of target RNA was determined from the appropriate standard curve and normalized relative to GAPDH mRNA. The assay was custom designed using the Primer Express Software 3.0 (Applied Biosystems).

### Immunostaining

IPSCs were fixed with 4% paraformaldehyde in PBS for 20 min at room temperature (16–25°C) and permeabilized in 0.2% Triton in PBS for 10 min at room temperature. Cells were then blocked by incubation for 20 min with 5% donkey serum in PBS at room temperature. Cells were washed three times with PBS before the addition of PBS containing antibodies against Nanog (1∶500, Rabbit polyclonal; ab21603, Abcam) and SSEA1 (1∶100, mouse monoclonal, MC80; Cell Signaling Technology). After incubation for 1 hour at room temperature, cells were then washed 3 times with PBS and labelled with Alexa 488- or Alexa 546-conjugated secondary antibodies (Invitrogen). After a final wash with PBS, a drop of Vectashield (containing DAPI) mounting solution was applied to the slide and a coverslip fitted. The samples were then analyzed using a LSM700 confocal microscope (Zeiss) according to manufacture's instructions.

### Bisulfide genomic sequencing

The promoters of *Oct4* and *Nanog* were analyzed using bisulfide genomic sequencing for the DNA methylation status of MEF, 4F-iPS, 25 iPSCs and ES cells. The bisulfide treatment was performed using the CpGenome modification kit (Qiagen) according to manufacture's instructions. The PCR primers for *Oct4* and *Nanog* were similar to those described previously by Takahashi and Yamanaka.

Oct4 F: ATTTGTTTTTTGGGTAGTTAAAGGT

Oct4 R: CCAACTATCTTCATCTTAATAACATCC

Nanog S: TGGTTAGGTTGGTTTTAAATTTTTG

Nanog AS: AACCCACCCTTATAAATTCTCAATTA

PCR products were cloned into pGEM T-vector (Promega) and sequenced using the M13 forward and reverse primers.

### Teratoma

iPSCs were. F1 mice of C57BL/6 and 129S5 were anesthetized with diethyl ether. 1×10^6^ iPSCs resuspended in PBS were administered subcutaneously into the dorsal flank. Tumours were visible 3 to 5 weeks after the injection, which were surgically dissected from the mice. Samples were weighed, fixed in PBS containing 4% formaldehyde, and embedded in paraffin. Sections were prepared and stained with hematoxylin and eosin.

### Microarray analysis

Total RNA from MEFs, ES cells or iPSCs were hybridized onto MouseWG-6 expression BeadChip (Illumina) according to manufacture's instructions. Arrays were then scanned using the BeadXpress Reader (Illumina). Raw expression files were exported directly from BeadStudio and loaded into R/Bioconductor [72]. The lumi package was used to perform Vanriance-stabilizing Transformation and Robust Spline Normalization on the expression values [73]. For analysis of differential expression, limma was used to fit linear models to every probe, using an empirical Bayes approach to shrink the estimated variance [74], [75]. Probes that passed a detection threshold <0.01 in at least one sample (these values are included in Illumina's BeadStudio output) were called ‘present’. To establish False Discovery Rates (FDR), the p-values of all the ‘present’ probes were adjusted using the Benjamini and Hochberg method. To highlight the similarities and differences between different cell types, a FDR of 10% was chosen as a cut-off point.

### Identification of potential miR-25 targets

The homozygous mutant cells were used for analysis as follows. *Dgcr8^gt1/tm1^* mouse embryonic stem cells were maintained in 100 µg/ml hygromycin B (Calbiochem) selective ES cell culture medium (500 ml GMEM (Sigma), 50 ml Foetal Bovine Serum (Gibco), 5 ml 200 mM L-Glutamine (Gibco), 5 ml 100 mM Sodium Pyruvate (Gibco), 5 ml 100× Non-Essential Amino Acids (Gibco), 70 µl 0.05 M ß-mercaptoethanol (Sigma) and LIF) in gelatinized plates. Prior to transfection the cells were cultured for 4 days in non-selective media. On the fourth day the cells were plated to gelatinised 6 well plates, 96×10^4^ cells per well in 7.2 ml of non-selective media. Wells were transfected after 3 hours. 240 pmoles of miRNA control mimic (miRIDIAN Negative Control #2 (Dharmacon CN-002000-01-05) or miRIDIAN mmu-miR-25 mimic (Dharmacon C-310564-01-0005)) were added to 240 µl OptiMEM I (Gibco). 7.2 µl of Lipofectamine 2000 (Invitrogen) was added to further 240 µl of OptiMEM and incubated for 5 minutes at room temperature. The two mixtures were subsequently combined. This mixture was incubated for 25 minutes at room temperature. Then the media in each well was replaced by 2.4 ml of fresh, non-selective media. The miRNA-lipid complexes were transferred to the wells and these were mixed gently. 5 hours later, the media was replaced with a further 7.2 ml of non-selective media. 10 hours after the initiation of transfection cells were washed twice with DPBS (-CaCl_2_ and MgCl_2_) (Gibco) and lysed with 1 ml of Trizol. Transfections were conducted in duplicate. Purified RNA was cleaned up with an RNeasy MiniElute Cleanup Kit (Qiagen) and labeled using the Total Prep RNA Amplification Kit (Illumina). 1500 ng of biotinylated RNA was hybridised to Illumina Mouse-6 v1.1 Expression Beadchips overnight at 58°C. The chips were washed, detected and scanned according to the manufacturers instructions and the scanner output was imported into Beadstudio v.3.1.8 (Illumina).

In addition Trizol was used to prepare RNA in triplicate from the 2 independently derived homozygous mutant mouse ES cell lines (*Dgcr8^gt1/tm1^* and *Dgcr8^gt2/tm1^*) and 2 corresponding heterozygous cell lines (*Dgcr8^tm1,gt1/+^* and *Dgcr8^tm1,gt2/+^*). The RNA was prepared for array analysis as described above.

Microarray analysis was conducted in R/Bioconductor [72] using the *lumi* [73] and *limma* [74] packages. All arrays relevant for this study were vst [75] transformed and quantile normalised alongside further array sets, the results of which will be discussed in a subsequent paper (Davis *et al* in preparation). The probes from the array were mapped to transcripts based upon the annotation of Ensembl v53. Where a probe maps to multiple transcripts, Vega [76] annotation was accepted in preference followed by Ensembl transcripts and EST transcripts respectively. In each category the transcript with the longest 3′UTR was chosen. If a probe was not associated with an Ensembl annotated transcript, where possible probes were annotated with a transcript according to the Illumina annotation file. Only probes with an annotated transcript were considered for the expression analysis. Where multiple probes mapped to the same transcript, the probe with the greatest inter-quartile range across the normalised arrays was selected for expression analysis. In order to determine a list of transcripts down-regulated by miR-25 a linear model was fitted across a large set of similar experiments prior to the comparison of the relevant samples (Davis *et al* in preparation); these include arrays considering a transfection time series and the transfection of alternative miRNAs. Arrays derived from *Dgcr8^gt1/tm1^* cells transfected with a miR-25 mimic, lysed 10 hours after transfection were compared to those derived from cells transfected with a control mimic lysed after the same interval. Probes down-regulated in the presence of the miRNA mimic with a log fold change greater than log_2_(1.2) and a p-value less than 0.1 were considered for further analysis.

The transcripts associated with these probes were mapped to further annotation based upon Ensembl version 53, where possible, or to EntrezIDs based upon the Ilumina annotation file if Ensembl annotation was unavailable. Sylamer [54] was used to count the number of miRNA seed sequences associated with miR-25 in the 3′UTR of the annotated transcripts. The final miRNA target list comprises of those transcripts from this potential target list with one or more 7mer[1A] or 7mer[2] seed sequence within their annotated 3′UTRs. This list was further refined through a comparison to those genes whose expression changed between heterozygote *Dgcr8* cells with two gene traps in the same allele of *Dgcr8* and homozygous mutant cells with a gene trap cassette in each allele of *Dgcr8*. A linear model was fitted across all of the heterozygous and homozygous mutant arrays and a fifth set incorporating wild-type ES cells (E14). Using the same set of refined probes described and considering each of the heterozygous lines and homozygous mutant lines with equal weight the *limma* [74] package in bioconductor [72] was used to determine the relative expression change for each probe and corresponding gene between the cell lines of differing genotype.

Microarray data in this study can be accessed at:

E-MTAB-409 <http://www.ebi.ac.uk/arrayexpress/experiments/E-MTAB-409>

E-MTAB-418 <http://www.ebi.ac.uk/arrayexpress/experiments/E-MTAB-418>

### Quantitation of miRNA

Quantitative real time PCR was performed using Taqman Gene Expression assays (Applied Biosystems) according to manufacture's instructions. The assay was custom designed using the Primer Express Software 3.0 (Applied Biosystems). The amount of target RNA was determined from the appropriate standard curve and normalized relative to *Gapdh* mRNA. Primer sequences are shown in Table S1. For mature miR-25 real time PCR analysis, we used a kit from the Taqman MicroRNA assays (Applied Biosystems). miR-25 expression was normalized against expression of Sno202 (Applied Biosystems).

### Sylamer target enrichment analysis

Sylamer utilizes the over- and under-representation P-values in nested bins across a ranked sequence universe. It is optimized for four letter alphabets (i.e. DNA/RNA) and computes P-values for all words of a fixed length. The method has been tested on different miRNA expression databases. A convenient post-processing step is to plot the Sylamer output as lines of log-transformed P-values across all bins, giving a comprehensive view of word distribution behaviour across the sequence universe.

### Luciferase plasmid construction

The Wwp2 3′UTR was amplified using primers Wwp2-F: 5′-TTTAACTCGAGCT-GAGGCTGCTGTCTCACAC-3′ and Wwp2-R: 5′-AAGCCGCGGCCGCGGCTGCTG-ATTCTTTATTGC-3′. The amplified fragment was digested using XhoI and NotI and ligated the psiCheck-2 renilla luciferase reporter plasmid (Promega). For the mutagenesis reactions, Quick Change Lightning Mutagenesis Kit (Strategne) was used. The mutagenic primers were Wwp2-mF: 5′-TCGGTATTGC-CAGTTTCTTGTAGACTAAAGAATCAGCAGC-3′ and Wwp2-mR: 5′-GCTGCT-GATTCTTTAGTCTACAAGAAACTGGCAATACCGA-3′.

Following the same cloning strategy, the Fbxw7 3′UTR was amplified using primers Fbxw7-F: 5′- TTTAACTCGAGAAAGCAGACATGATGAATTTTG-3′ and Fbxw7-R: 5′– AAGCCGCGGCCGCTAACATGAAAAAACACATTTTAT-3′. For the mutagenesis reactions, the following primers were used: Fbxw7-mF1: 5′ – GACGACTC-TCTAAATCCAACCAGGTGGAATTATTCTTTG-3′ and Fbxw7-mR1: 5′ – CAAAG-AATAATTCCACCTGGTTGGATTTAGAGAGTCGTC-3′ for site 1. Fbxw7-mF2: 5′- CTTATAACTTAAGTGGAATAAAATGTGTTTTTTCATGTTAGCGGC-3′ and Fbxw7-mR2: 5′-GCCGCTAACATGAAAAAACACATTTTATTCCACTTAAG-TTATAAG-3′ for site 2.

All plasmids were verified by sequencing.

### Luciferase assays

Luciferase reactions were performed in 96 well plates. HeLaS3 cells were transfected using the Lipofectamin 2000 reagents (Invitrogen) with 5 ng of the Wwp2 or the Fbxw7 3′UTR plasmids. This value was derived after a concentration curve of the plamid was tested. A miR-25 mimic (Dharmacon) was added at a concentration of 20 nM. Different concentrations were tested and 20 nM was chosen as the lowest concentration to cause significant inhibition (data not shown). Reporter activity was analysed 24 hours after transfection with the Dual-Luciferase Reporter Assay System (Promega). Expression data are given as a ratio, with the Renilla reporter normalized against the luciferase reporter values.

### Data analysis

Data are presented as mean ±SEM. Comparison between groups were performed using one-way ANOVA followed by the Newman-Keuls post test to determine statistical significance. A P value of <0.05 was taken as statistically significant.

## Supporting Information

Table S1 **miRNAs used in the screen.**
(XLS)

Table S2 **PCR primers for amplifying miRNA genomic DNA fragments and for other experiments in this work.**
(PDF)

Table S3 **Conservation of miR-25 and miR-298 mature sequences.** All mature sequences of miR-25 and miR-298 are obtained from miRBase database.
(PDF)

Table S4 **Candidate target genes of miR-25.** The table lists 54 genes based on microarray expression and Sylamer analysis following transfection of miR-25 mimic to the *Dgcr8-*deficient ES cells.
(PDF)

## References

[1] Takahashi K, Yamanaka S (2006) Induction of pluripotent stem cells from mouse embryonic and adult fibroblast cultures by defined factors. Cell 126: 663–676. 16904174 10.1016/j.cell.2006.07.024

[2] Yu J, Vodyanik MA, Smuga-Otto K, Antosiewicz-Bourget J, Frane JL, et al (2007) Induced pluripotent stem cell lines derived from human somatic cells. Science 318: 1917–1920. 18029452 10.1126/science.1151526

[3] Okita K, Ichisaka T, Yamanaka S (2007) Generation of germline-competent induced pluripotent stem cells. Nature 448: 313–317. 17554338 10.1038/nature05934

[4] Wernig M, Meissner A, Foreman R, Brambrink T, Ku M, et al (2007) In vitro reprogramming of fibroblasts into a pluripotent ES-cell-like state. Nature 448: 318–324. 17554336 10.1038/nature05944

[5] Takahashi K, Tanabe K, Ohnuki M, Narita M, Ichisaka T, et al (2007) Induction of pluripotent stem cells from adult human fibroblasts by defined factors. Cell 131: 861–872. 18035408 10.1016/j.cell.2007.11.019

[6] Lowry WE, Richter L, Yachechko R, Pyle AD, Tchieu J, et al (2008) Generation of human induced pluripotent stem cells from dermal fibroblasts. Proc Natl Acad Sci U S A 105: 2883–2888. 18287077 10.1073/pnas.0711983105 PMC2268554

[7] Park IH, Zhao R, West JA, Yabuuchi A, Huo H, et al (2008) Reprogramming of human somatic cells to pluripotency with defined factors. Nature 451: 141–146. 18157115 10.1038/nature06534

[8] Aoi T, Yae K, Nakagawa M, Ichisaka T, Okita K, et al (2008) Generation of pluripotent stem cells from adult mouse liver and stomach cells. Science 321: 699–702. 18276851 10.1126/science.1154884

[9] Hanna J, Markoulaki S, Schorderet P, Carey BW, Beard C, et al (2008) Direct reprogramming of terminally differentiated mature B lymphocytes to pluripotency. Cell 133: 250–264. 18423197 10.1016/j.cell.2008.03.028 PMC2615249

[10] Stadtfeld M, Brennand K, Hochedlinger K (2008) Reprogramming of pancreatic beta cells into induced pluripotent stem cells. Curr Biol 18: 890–894. 18501604 10.1016/j.cub.2008.05.010 PMC2819222

[11] Aasen T, Raya A, Barrero MJ, Garreta E, Consiglio A, et al (2008) Efficient and rapid generation of induced pluripotent stem cells from human keratinocytes. Nat Biotechnol 26: 1276–1284. 18931654 10.1038/nbt.1503

[12] Kim JB, Zaehres H, Wu G, Gentile L, Ko K, et al (2008) Pluripotent stem cells induced from adult neural stem cells by reprogramming with two factors. Nature 454: 646–650. 18594515 10.1038/nature07061

[13] Stadtfeld M, Nagaya M, Utikal J, Weir G, Hochedlinger K (2008) Induced pluripotent stem cells generated without viral integration. Science 322: 945–949. 18818365 10.1126/science.1162494 PMC3987909

[14] Okita K, Nakagawa M, Hyenjong H, Ichisaka T, Yamanaka S (2008) Generation of mouse induced pluripotent stem cells without viral vectors. Science 322: 949–953. 18845712 10.1126/science.1164270

[15] Yu J, Hu K, Smuga-Otto K, Tian S, Stewart R, et al (2009) Human induced pluripotent stem cells free of vector and transgene sequences. Science 324: 797–801. 19325077 10.1126/science.1172482 PMC2758053

[16] Woltjen K, Michael IP, Mohseni P, Desai R, Mileikovsky M, et al (2009) piggyBac transposition reprograms fibroblasts to induced pluripotent stem cells. Nature 458: 766–770. 19252478 10.1038/nature07863 PMC3758996

[17] Yusa K, Rad R, Takeda J, Bradley A (2009) Generation of transgene-free induced pluripotent mouse stem cells by the piggyBac transposon. Nat Methods 6: 363–369. 19337237 10.1038/nmeth.1323 PMC2677165

[18] Han J, Yuan P, Yang H, Zhang J, Soh BS, et al (2010) Tbx3 improves the germ-line competency of induced pluripotent stem cells. Nature 463: 1096–1100. 20139965 10.1038/nature08735 PMC2901797

[19] Heng JC, Feng B, Han J, Jiang J, Kraus P, et al (2010) The nuclear receptor Nr5a2 can replace Oct4 in the reprogramming of murine somatic cells to pluripotent cells. Cell Stem Cell 6: 167–174. 20096661 10.1016/j.stem.2009.12.009

[20] Mali P, Ye Z, Hommond HH, Yu X, Lin J, et al (2008) Improved efficiency and pace of generating induced pluripotent stem cells from human adult and fetal fibroblasts. Stem Cells 26: 1998–2005. 18511599 10.1634/stemcells.2008-0346

[21] Shi Y, Do JT, Desponts C, Hahm HS, Scholer HR, et al (2008) A combined chemical and genetic approach for the generation of induced pluripotent stem cells. Cell Stem Cell 2: 525–528. 18522845 10.1016/j.stem.2008.05.011

[22] Huangfu D, Maehr R, Guo W, Eijkelenboom A, Snitow M, et al (2008) Induction of pluripotent stem cells by defined factors is greatly improved by small-molecule compounds. Nat Biotechnol 26: 795–797. 18568017 10.1038/nbt1418 PMC6334647

[23] Silva J, Barrandon O, Nichols J, Kawaguchi J, Theunissen TW, et al (2008) Promotion of reprogramming to ground state pluripotency by signal inhibition. PLoS Biol 6: e253. 18942890 10.1371/journal.pbio.0060253 PMC2570424

[24] Marson A, Foreman R, Chevalier B, Bilodeau S, Kahn M, et al (2008) Wnt signaling promotes reprogramming of somatic cells to pluripotency. Cell Stem Cell 3: 132–135. 18682236 10.1016/j.stem.2008.06.019 PMC3235673

[25] Hong H, Takahashi K, Ichisaka T, Aoi T, Kanagawa O, et al (2009) Suppression of induced pluripotent stem cell generation by the p53-p21 pathway. Nature 460: 1132–1135. 19668191 10.1038/nature08235 PMC2917235

[26] Li H, Collado M, Villasante A, Strati K, Ortega S, et al (2009) The Ink4/Arf locus is a barrier for iPS cell reprogramming. Nature 460: 1136–1139. 19668188 10.1038/nature08290 PMC3578184

[27] Kawamura T, Suzuki J, Wang YV, Menendez S, Morera LB, et al (2009) Linking the p53 tumour suppressor pathway to somatic cell reprogramming. Nature 460: 1140–1144. 19668186 10.1038/nature08311 PMC2735889

[28] Utikal J, Polo JM, Stadtfeld M, Maherali N, Kulalert W, et al (2009) Immortalization eliminates a roadblock during cellular reprogramming into iPS cells. Nature 460: 1145–1148. 19668190 10.1038/nature08285 PMC3987892

[29] Bartel DP (2009) MicroRNAs: target recognition and regulatory functions. Cell 136: 215–233. 19167326 10.1016/j.cell.2009.01.002 PMC3794896

[30] Lee Y, Ahn C, Han J, Choi H, Kim J, et al (2003) The nuclear RNase III Drosha initiates microRNA processing. Nature 425: 415–419. 14508493 10.1038/nature01957

[31] Denli AM, Tops BB, Plasterk RH, Ketting RF, Hannon GJ (2004) Processing of primary microRNAs by the Microprocessor complex. Nature 432: 231–235. 15531879 10.1038/nature03049

[32] Gregory RI, Yan KP, Amuthan G, Chendrimada T, Doratotaj B, et al (2004) The Microprocessor complex mediates the genesis of microRNAs. Nature 432: 235–240. 15531877 10.1038/nature03120

[33] Han J, Lee Y, Yeom KH, Nam JW, Heo I, et al (2006) Molecular basis for the recognition of primary microRNAs by the Drosha-DGCR8 complex. Cell 125: 887–901. 16751099 10.1016/j.cell.2006.03.043

[34] Grishok A, Pasquinelli AE, Conte D, Li N, Parrish S, et al (2001) Genes and mechanisms related to RNA interference regulate expression of the small temporal RNAs that control C. elegans developmental timing. Cell 106: 23–34. 11461699 10.1016/s0092-8674(01)00431-7

[35] Ketting RF, Fischer SE, Bernstein E, Sijen T, Hannon GJ, et al (2001) Dicer functions in RNA interference and in synthesis of small RNA involved in developmental timing in C. elegans. Genes Dev 15: 2654–2659. 11641272 10.1101/gad.927801 PMC312808

[36] Kanellopoulou C, Muljo SA, Kung AL, Ganesan S, Drapkin R, et al (2005) Dicer-deficient mouse embryonic stem cells are defective in differentiation and centromeric silencing. Genes Dev 19: 489–501. 15713842 10.1101/gad.1248505 PMC548949

[37] Murchison EP, Partridge JF, Tam OH, Cheloufi S, Hannon GJ (2005) Characterization of Dicer-deficient murine embryonic stem cells. Proc Natl Acad Sci U S A 102: 12135–12140. 16099834 10.1073/pnas.0505479102 PMC1185572

[38] Wang Y, Medvid R, Melton C, Jaenisch R, Blelloch R (2007) DGCR8 is essential for microRNA biogenesis and silencing of embryonic stem cell self-renewal. Nat Genet 39: 380–385. 17259983 10.1038/ng1969 PMC3008549

[39] Wang Y, Baskerville S, Shenoy A, Babiarz JE, Baehner L, et al (2008) Embryonic stem cell-specific microRNAs regulate the G1-S transition and promote rapid proliferation. Nat Genet 40: 1478–1483. 18978791 10.1038/ng.250 PMC2630798

[40] Marson A, Levine SS, Cole MF, Frampton GM, Brambrink T, et al (2008) Connecting microRNA genes to the core transcriptional regulatory circuitry of embryonic stem cells. Cell 134: 521–533. 18692474 10.1016/j.cell.2008.07.020 PMC2586071

[41] Judson RL, Babiarz JE, Venere M, Blelloch R (2009) Embryonic stem cell-specific microRNAs promote induced pluripotency. Nat Biotechnol 27: 459–461. 19363475 10.1038/nbt.1535 PMC2743930

[42] Lin SL, Chang DC, Chang-Lin S, Lin CH, Wu DT, et al (2008) Mir-302 reprograms human skin cancer cells into a pluripotent ES-cell-like state. Rna 14: 2115–2124. 18755840 10.1261/rna.1162708 PMC2553732

[43] Anokye-Danso F, Trivedi CM, Juhr D, Gupta M, Cui Z, et al (2011) Highly efficient miRNA-mediated reprogramming of mouse and human somatic cells to pluripotency. Cell Stem Cell 8: 376–388. 21474102 10.1016/j.stem.2011.03.001 PMC3090650

[44] Miyoshi N, Ishii H, Nagano H, Haraguchi N, Dewi DL, et al (2011) Reprogramming of mouse and human cells to pluripotency using mature microRNAs. Cell stem cell 8: 633–638. 21620789 10.1016/j.stem.2011.05.001

[45] Melton C, Judson RL, Blelloch R (2010) Opposing microRNA families regulate self-renewal in mouse embryonic stem cells. Nature 463: 621–626. 20054295 10.1038/nature08725 PMC2894702

[46] Houbaviy HB, Murray MF, Sharp PA (2003) Embryonic stem cell-specific MicroRNAs. Dev Cell 5: 351–358. 12919684 10.1016/s1534-5807(03)00227-2

[47] Tang F, Hajkova P, Barton SC, Lao K, Surani MA (2006) MicroRNA expression profiling of single whole embryonic stem cells. Nucleic Acids Res 34: e9. 16434699 10.1093/nar/gnj009 PMC1351374

[48] Rosenfeld N, Aharonov R, Meiri E, Rosenwald S, Spector Y, et al (2008) MicroRNAs accurately identify cancer tissue origin. Nat Biotechnol 26: 462–469. 18362881 10.1038/nbt1392

[49] Lu J, Getz G, Miska EA, Alvarez-Saavedra E, Lamb J, et al (2005) MicroRNA expression profiles classify human cancers. Nature 435: 834–838. 15944708 10.1038/nature03702

[50] Wang W, Lin C, Lu D, Ning Z, Cox T, et al (2008) Chromosomal transposition of PiggyBac in mouse embryonic stem cells. Proc Natl Acad Sci U S A 105: 9290–9295. 18579772 10.1073/pnas.0801017105 PMC2440425

[51] Wang W, Yang J, Liu H, Lu D, Chen X, et al (2011) Rapid and efficient reprogramming of somatic cells to induced pluripotent stem cells by retinoic acid receptor gamma and liver receptor homolog 1. Proceedings of the National Academy of Sciences of the United States of America 108: 18283–18288. 21990348 10.1073/pnas.1100893108 PMC3215025

[52] Niwa H, Miyazaki J, Smith AG (2000) Quantitative expression of Oct-3/4 defines differentiation, dedifferentiation or self-renewal of ES cells. Nat Genet 24: 372–376. 10742100 10.1038/74199

[53] Ying QL, Wray J, Nichols J, Batlle-Morera L, Doble B, et al (2008) The ground state of embryonic stem cell self-renewal. Nature 453: 519–523. 18497825 10.1038/nature06968 PMC5328678

[54] van Dongen S, Abreu-Goodger C, Enright AJ (2008) Detecting microRNA binding and siRNA off-target effects from expression data. Nat Methods 5: 1023–1025. 18978784 10.1038/nmeth.1267 PMC2635553

[55] Xu H, Wang W, Li C, Yu H, Yang A, et al (2009) WWP2 promotes degradation of transcription factor OCT4 in human embryonic stem cells. Cell Res 19: 561–573. 19274063 10.1038/cr.2009.31

[56] Xu HM, Liao B, Zhang QJ, Wang BB, Li H, et al (2004) Wwp2, an E3 ubiquitin ligase that targets transcription factor Oct-4 for ubiquitination. J Biol Chem 279: 23495–23503. 15047715 10.1074/jbc.M400516200

[57] Yada M, Hatakeyama S, Kamura T, Nishiyama M, Tsunematsu R, et al (2004) Phosphorylation-dependent degradation of c-Myc is mediated by the F-box protein Fbw7. Embo J 23: 2116–2125. 15103331 10.1038/sj.emboj.7600217 PMC424394

[58] Koepp DM, Schaefer LK, Ye X, Keyomarsi K, Chu C, et al (2001) Phosphorylation-dependent ubiquitination of cyclin E by the SCFFbw7 ubiquitin ligase. Science 294: 173–177. 11533444 10.1126/science.1065203

[59] Minella AC, Clurman BE (2005) Mechanisms of tumor suppression by the SCF(Fbw7). Cell Cycle 4: 1356–1359. 16131838 10.4161/cc.4.10.2058

[60] Welcker M, Clurman BE (2008) FBW7 ubiquitin ligase: a tumour suppressor at the crossroads of cell division, growth and differentiation. Nat Rev Cancer 8: 83–93. 18094723 10.1038/nrc2290

[61] Liu N, Li H, Li S, Shen M, Xiao N, et al (2010) The Fbw7/hCDC4 tumor suppressor targets pro-proliferative factor KLF5 for ubiquitination and degradation through multiple phosphodegron motifs. J Biol Chem 10.1074/jbc.M109.099440 PMC2881808 20388706

[62] Jiang J, Chan YS, Loh YH, Cai J, Tong GQ, et al (2008) A core Klf circuitry regulates self-renewal of embryonic stem cells. Nat Cell Biol 10: 353–360. 18264089 10.1038/ncb1698

[63] Feng B, Jiang J, Kraus P, Ng JH, Heng JC, et al (2009) Reprogramming of fibroblasts into induced pluripotent stem cells with orphan nuclear receptor Esrrb. Nat Cell Biol 11: 197–203. 19136965 10.1038/ncb1827

[64] Kumar M, Lu Z, Takwi AA, Chen W, Callander NS, et al (2011) Negative regulation of the tumor suppressor p53 gene by microRNAs. Oncogene 30: 843–853. 20935678 10.1038/onc.2010.457 PMC3021102

[65] Li Z, Yang CS, Nakashima K, Rana TM (2011) Small RNA-mediated regulation of iPS cell generation. The EMBO journal 30: 823–834. 21285944 10.1038/emboj.2011.2 PMC3049216

[66] Zhao Y, Yin X, Qin H, Zhu F, Liu H, et al (2008) Two supporting factors greatly improve the efficiency of human iPSC generation. Cell stem cell 3: 475–479. 18983962 10.1016/j.stem.2008.10.002

[67] Li R, Liang J, Ni S, Zhou T, Qing X, et al (2010) A mesenchymal-to-epithelial transition initiates and is required for the nuclear reprogramming of mouse fibroblasts. Cell stem cell 7: 51–63. 20621050 10.1016/j.stem.2010.04.014

[68] Brett JO, Renault VM, Rafalski VA, Webb AE, Brunet A (2011) The microRNA cluster miR-106b∼25 regulates adult neural stem/progenitor cell proliferation and neuronal differentiation. Aging 3: 108–124. 21386132 10.18632/aging.100285 PMC3082007

[69] Razumilava N, Bronk SF, Smoot RL, Fingas CD, Werneburg NW, et al (2012) miR-25 targets TNF-related apoptosis inducing ligand (TRAIL) death receptor-4 and promotes apoptosis resistance in cholangiocarcinoma. Hepatology 55: 465–475. 21953056 10.1002/hep.24698 PMC3268937

[70] Zhang H, Zuo Z, Lu X, Wang L, Wang H, et al (2012) MiR-25 regulates apoptosis by targeting Bim in human ovarian cancer. Oncology reports 27: 594–598. 22076535 10.3892/or.2011.1530

[71] Anton R, Chatterjee SS, Simundza J, Cowin P, Dasgupta R (2011) A systematic screen for micro-RNAs regulating the canonical Wnt pathway. PloS one 6: e26257. 22043311 10.1371/journal.pone.0026257 PMC3197157

[72] Gentleman RC, Carey VJ, Bates DM, Bolstad B, Dettling M, et al (2004) Bioconductor: open software development for computational biology and bioinformatics. Genome biology 5: R80. 15461798 10.1186/gb-2004-5-10-r80 PMC545600

[73] Du P, Kibbe WA, Lin SM (2008) lumi: a pipeline for processing Illumina microarray. Bioinformatics 24: 1547–1548. 18467348 10.1093/bioinformatics/btn224

[74] Smyth GK (2004) Linear models and empirical bayes methods for assessing differential expression in microarray experiments. Statistical applications in genetics and molecular biology 3: Article3. 16646809 10.2202/1544-6115.1027

[75] Lin SM, Du P, Huber W, Kibbe WA (2008) Model-based variance-stabilizing transformation for Illumina microarray data. Nucleic acids research 36: e11. 18178591 10.1093/nar/gkm1075 PMC2241869

[76] Wilming LG, Gilbert JG, Howe K, Trevanion S, Hubbard T, et al (2008) The vertebrate genome annotation (Vega) database. Nucleic acids research 36: D753–760. 18003653 10.1093/nar/gkm987 PMC2238886

[77] Gu P, Reid JG, Gao X, Shaw CA, Creighton C, et al (2008) Novel microRNA candidates and miRNA-mRNA pairs in embryonic stem (ES) cells. PloS one 3: e2548. 18648548 10.1371/journal.pone.0002548 PMC2481296

[78] Hayashi K, Chuva de Sousa Lopes SM, Kaneda M, Tang F, Hajkova P, et al (2008) MicroRNA biogenesis is required for mouse primordial germ cell development and spermatogenesis. PloS one 3: e1738. 18320056 10.1371/journal.pone.0001738 PMC2254191

[79] Mi S, Lu J, Sun M, Li Z, Zhang H, et al (2007) MicroRNA expression signatures accurately discriminate acute lymphoblastic leukemia from acute myeloid leukemia. Proceedings of the National Academy of Sciences of the United States of America 104: 19971–19976. 18056805 10.1073/pnas.0709313104 PMC2148407

[80] Shen J, Ambrosone CB, DiCioccio RA, Odunsi K, Lele SB, et al (2008) A functional polymorphism in the miR-146a gene and age of familial breast/ovarian cancer diagnosis. Carcinogenesis 29: 1963–1966. 18660546 10.1093/carcin/bgn172

[81] Zhang L, Huang J, Yang N, Greshock J, Megraw MS, et al (2006) microRNAs exhibit high frequency genomic alterations in human cancer. Proceedings of the National Academy of Sciences of the United States of America 103: 9136–9141. 16754881 10.1073/pnas.0508889103 PMC1474008

[82] Yanaihara N, Caplen N, Bowman E, Seike M, Kumamoto K, et al (2006) Unique microRNA molecular profiles in lung cancer diagnosis and prognosis. Cancer cell 9: 189–198. 16530703 10.1016/j.ccr.2006.01.025

[83] Peltier HJ, Latham GJ (2008) Normalization of microRNA expression levels in quantitative RT-PCR assays: identification of suitable reference RNA targets in normal and cancerous human solid tissues. RNA 14: 844–852. 18375788 10.1261/rna.939908 PMC2327352

[84] Barroso-delJesus A, Romero-Lopez C, Lucena-Aguilar G, Melen GJ, Sanchez L, et al (2008) Embryonic stem cell-specific miR302-367 cluster: human gene structure and functional characterization of its core promoter. Molecular and cellular biology 28: 6609–6619. 18725401 10.1128/MCB.00398-08 PMC2573233

