# Supplementary material for: MiR-25 Regulates Wwp2 and Fbxw7 and Promotes Reprogramming of Mouse Fibroblast Cells to iPSCs
Source: PLoS One. 2012 Aug 17;7(8):e40938. doi: 10.1371/journal.pone.0040938 (PMC3422229; doi:10.1371/journal.pone.0040938)
Supplement: Table S2 — PCR primers for amplifying miRNA genomic DNA fragments and for other experiments in this work. (PDF) [file pone.0040938.s002.pdf]

Table S2. PCR primers for amplifying miRNA from DNA and RT-PCR/real time PCR primers

No.	Name	Primers	Description
1	miR-10b	CTAGCCTGCAGGATCCTGGCAAGCCGATGAGGG	miR-10b Amplification Primers
		ATCCGGCCGGCCCCTTTCACCTCACTCTCTAAT	
2	miR-16-1	CTAGCCTGCAGGGATCTAGGATTACAAGTGTACC	miR-16-1 Amplification Primers
		ATCCGGCCGGCCATTATGCACATACCAGTGTTAG	
3	miR-18a	CTAGCCTGCAGGAGCTGTAATTGATGTTTGTG	miR-18a Amplification Primers
		ATCCGGCCGGCCGCAGTACTTTAAGTGCTCGT	
4	miR-19a	CTAGCCTGCAGGCAGGTAGTGATGTGTGCATC	miR-19a Amplification Primers
		ATCCGGCCGGCCTGGATTTGCACAGCAGAATA	
5	miR-19b-1	CTAGCCTGCAGGGTTGTGTTTGCAGCCCTCTG	miR-19b-1 Amplification Primers
		ATCCGGCCGGCCAACAAACCACAATTTAAAAA	
6	miR-24-1	CTAGCCTGCAGGCGTGCTGACCACCCTTAAGT	miR-24-1 Amplification Primers
		ATCCGGCCGGCCGAAAGGCTCTACAGACAAGG	
7	miR-25/93	CTAGCCTGCAGGTTGCTGCTTGAATCCATGAG	miR-25/93 Amplification Primers
		ATCCGGCCGGCCGTCCAGTCTCACTCTTCATT	
8	miR-29a	CTAGCCTGCAGGTAGCACCATTTGAAATCAGT	miR-29a Amplification Primers
		ATCCGGCCGGCCGTGAGCTGTTAGAACCTTAT	
9	miR-29b-2	CTAGCCTGCAGGATTGTCATTGTCTTGAACAA	miR-29b-2 Amplification Primers
		ATCCGGCCGGCCGTGAATGAAGCAGTCCTCCA	
10	miR-29c	CTAGCCTGCAGGAGGCAGTGATAGTGAGAAAG	miR-29c Amplification Primers
		ATCCGGCCGGCCAAAAATAGTAGATAAAACAG	
11	miR-30a	CTAGCCTGCAGGATAAACTTACTCATGTTCTA	miR-30a Amplification Primers
		ATCCGGCCGGCCTACTCTGAGATTTGATAAAT	
12	miR-30b	CTAGCCTGCAGGTAGGTGGGAAAAGCTATAGA	miR-30b Amplification Primers
		ATCCGGCCGGCCGCTGACAAAGAAGTGGAGAC	
13	miR-30c-1	CTAGCCTGCAGGGACAGTTTGTCATGCAAGTA	miR-30c-1 Amplification Primers
		ATCCGGCCGGCCGCGATGGAAGATGCTACCCA	
14	miR-30e	CTAGCCTGCAGGGGTTTAGTGTAATATGCCTC	miR-30e Amplification Primers
		ATCCGGCCGGCCTCTCTTTAGTCATCTATTCA	
15	miR-31	CTAGCCTGCAGGTATGGACTATGAAAATGGCT	miR-31 Amplification Primers
		ATCCGGCCGGCCTTCTGACAAGTCAGAGCAGG	
16	miR-32	CTAGCCTGCAGGGAGTGAGATTGAGAGTTAGT	miR-32 Amplification Primers
		ATCCGGCCGGCCCTTTAAACAAGTATTACTTA	
17	miR-93	CTAGCCTGCAGGGGTGAGTGGTGGGTCCCTGT	miR-93 Amplification Primers
		ATCCGGCCGGCCCTTTCTTTGCTCCAGCTTCA	
18	miR-100	CTAGCCTGCAGGCAGGCACTTGTTCTATCTCT	miR-100 Amplification Primers
		ATCCGGCCGGCCTTTTGTATGAGCCTATTTAT	
19	miR-106b	CTAGCCTGCAGGGCCTGCTTTCCCGCTTTCCC	miR-106b Amplification Primers
		ATCCGGCCGGCCGAGACCAGACCCTTCTGAAC	
20	miR-124a-1	CTAGCCTGCAGGAGCTGCGGTAGGAAGGATGC	miR-124a-1 Amplification Primers
		ATCCGGCCGGCCCAGGCTATTTTCTGCCGCCG	
21	miR-124a-2	CTAGCCTGCAGGACTTCGATCCATGTCATCAT	miR-124a-2 Amplification Primers
		ATCCGGCCGGCCTCCGTTTTGTTTTCCCAGTT	
22	miR-125a	CTAGCCTGCAGGATAGGAGCTGGGGTGTCTTC	miR-125a Amplification Primers
		ATCCGGCCGGCCTGCCCACAAACAGCTGGCAGA	
23	miR-126	CTAGCCTGCAGGACAGCAGGTAAACTTGCCTT	miR-126 Amplification Primers
		ATCCGGCCGGCCCCTGTTCTAGCACATCAACC	
24	miR-127	CTAGCCTGCAGGAAGCAGCTCCAGTTTTGTGAAC	miR-127 Amplification Primers
		ATCCGGCCGGCCCTTCTCCATCGTCTTAGGCAT	
25	miR-128-1	CTAGCCTGCAGGTGTCTTACTGTTAATAACTG	miR-128-1 Amplification Primers
		ATCCGGCCGGCCAGTAATGAGTTTGGCATGCT	
26	miR-146a	CTAGCCTGCAGGCTGCCCTTGGACCAGCAGTC	miR-146a Amplification Primers
		ATCCGGCCGGCCGCTCTCTTTTTCTTCTTGAC	

Table S2. PCR primers for amplifying miRNA from DNA and RT-PCR/real time PCR primers

27	miR-151	CTAGCCTGCAGGTCTAGGAAAGTATTGCTGCT	miR-151 Amplification Primers
		ATCCGGCCGGCCAAGGGGACAAATTTGGGGAT	
28	miR-155	CTAGCCTGCAGGTATTCAAATATTTCCACAGA	miR-155 Amplification Primers
		ATCCGGCCGGCCTGAAGATGGTTATGAACATA	
29	miR-181a-1	CTAGCCTGCAGGCCTGCTTCTTTTCTTCTGTA	miR-181a-1 Amplification Primers
		ATCCGGCCGGCCCTTTGGTTCTTCCTCCCACC	
30	miR-181a-2	CTAGCCTGCAGGTACAAATCCTACTACACATA	miR-181a-2 Amplification Primers
		ATCCGGCCGGCCAGATTTTTTTCCTACAGTAA	
31	miR-191	CTAGCCTGCAGGCTTCCTCTCGGGTCTCACGG	miR-191 Amplification Primers
		ATCCGGCCGGCCCCATGAAGACGGTTGGGGTGG	
32	miR-199b	CTAGCCTGCAGGAACCCATCTAGGGAGGCCCG	miR-199b Amplification Primers
		ATCCGGCCGGCCTGTGCAGGGGTCCCATCAGC	
33	miR-290	CTAGCCTGCAGGGCTAGGAGTTACTGAGGGCAG	miR-290 Amplification Primers
		ATCCGGCCGGCCCCGCTACATAGGTGTAACAGCT	
34	miR-291a	CTAGCCTGCAGGACGGCTATCTGGCACATTTAC	miR-291a Amplification Primers
		ATCCGGCCGGCCCAAAAGAGCCCCCAGTTTGAG	
35	miR-292	CTAGCCTGCAGGAAGTGCTTCCACTTTGTGTGC	miR-292 Amplification Primers
		ATCCGGCCGGCCATCGACACTGTATGTTCCAAC	
36	miR-293	CTAGCCTGCAGGCAAGCTTGGTGGACAGCCGG	miR-293 Amplification Primers
		ATCCGGCCGGCCTCCACTTAAAAGCTTAGATAG	
37	miR-294	CTAGCCTGCAGGTTGCCGATTGAGAAACTGCAG	miR-294 Amplification Primers
		ATCCGGCCGGCCCACCTTGGTTCATTCAATAGCA	
38	miR-295	CTAGCCTGCAGGTACTGTATTACATATAGCTGC	miR-295 Amplification Primers
		ATCCGGCCGGCCAGGGTGCTTGCATGCTTGCAAG	
39	miR-298	CTAGCCTGCAGGTTCCCAGCTACGTGCGCTCAG	miR-298 Amplification Primers
		ATCCGGCCGGCCTACAGGATACTTGCCACACCAT	
40	miR-301a	CTAGCCTGCAGGGATGCCCATGTTAGCTACTAAA	miR-301a Amplification Primers
		ATCCGGCCGGCCCAAAGTACTATTCAGTTAGACA	
41	miR-301b	CTAGCCTGCAGGTGGCTGGAGTGGGATCTTAGA	miR-301b Amplification Primers
		ATCCGGCCGGCCGGCCTCTCTCCTTTGTGTTTGA	
42	miR-302a	CTAGCCTGCAGGTCGAAGAGTAAGTGCTTCCATG	miR-302a Amplification Primers
		ATCCGGCCGGCCGTGCAATTCCAATTTCTATTCA	
43	miR-302c	CTAGCCTGCAGGGGTAAAGCTTATGTCTGTAACC	miR-302c Amplification Primers
		ATCCGGCCGGCCAATGCACAGCAAGTGCCTCCA	
44	miR-302cluste	CTAGCCTGCAGGGTCTTGTAATGATTCTCAGAGA	miR-302cluster Amplification Primers
		ATCCGGCCGGCCACTTAGAATTCTGCTCTACTGA	
45	miR-363	CTAGCCTGCAGG GATTTCTGAGTTCGAGG	miR-363 Amplification Primers
		ATCCGGCCGGCCCATCCATGCCCATTCATCCA	
46	miR-367	CTAGCCTGCAGGGTTATTGACTGGGCTTCCCACC	miR-367 Amplification Primers
		ATCCGGCCGGCCGAGGTACCGTGGCAGTAGGGT	
47	miR-let7a-1	CTAGCCTGCAGGCAAGAAAGGTTAACATTAAATC	miR-let7a-1 Amplification Primers
		ATCCGGCCGGCCATTGAATTAGAGGCTTATAGCC	
48	miR-let7a-2	CTAGCCTGCAGGCTTGCATGAATACAAAATTTCA	miR-let7a-2 Amplification Primers
		ATCCGGCCGGCCTGTGTTGCTCATTATGCAGCAT	
49	miR-let7c-1	CTAGCCTGCAGGGCTGCTAATGGAAGTGTGGTC	miR-let7c-1 Amplification Primers
		ATCCGGCCGGCCTAATGCAACTTAATTCACTAC	
50	miR-let7c-2	CTAGCCTGCAGGCCCATTGTGGCCATTCCTGTG	miR-let7c-2 Amplification Primers
		ATCCGGCCGGCCGGCAGGAAGGCCCCTTCAGTG	
51	miR-let7e	CTAGCCTGCAGGGGTCTGGATCTCTGGCCTTGA	miR-let7e Amplification Primers
		ATCCGGCCGGCCCTAGAGAAACAGAAGCTTCT	
52	miR-let7g	CTAGCCTGCAGGTACTAGAATTCTGAGGGTATA	miR-let7g Amplification Primers
		ATCCGGCCGGCCAATGAGAGGGCATTTGCAATA	
53	Oct4-cMyc	AAGATCAAGTATTGAGTATTCC	Junction Primers

Table S2. PCR primers for amplifying miRNA from DNA and RT-PCR/real time PCR primers

		GCTGGTGATAGAAATTCTCTTCCT	
54	cMyc-Klf4	CTGTCCATTCAAGCAGACGAGC	Junction Primers
		TTACTGCTGCAAGCTGCACCAG	
55	Klf4-Sox2	CCGCAAACACACAGGGCACCGG	Junction Primers
		CTTGCTGATCTCCGAGTTGTG	
56	Fgf4	CGTGGTGAGCATCTTCGGAGTGG	RT-PCR for Fgf4
		CCTTCTTGGTCCGCCCGTTCTTA	
57	Klf4	CACCATGGACCCGGGCGTGGCTGCCAGAAA	RT-PCR for Klf4
		TTAGGCTGTTCTTTTCCGGGGCCACGA	
58	Fbx15	GTTGGAATCTGCTTCTACAG	RT-PCR for Fbx15
		CTTCACCAAGATTTCCGATG	
59	Sox2	GGTTACCTCTTCCTCCCACTCCAG	RT-PCR for Sox2
		TCACATGTGCGACAGGGGCAG	
60	Oct3/4	CTGAGGGCCAGGCAGGAGCACGAG	RT-PCR for Oct3/4
		CTGTAGGGAGGGCTTCGGGCACTT	
61	Zfp296	CCATTAGGGGCCATCATCGCTTTC	RT-PCR for Zfp296
		CACTGCTCACTGGAGGGGGCTTGC	
62	Gdf3	GTTCCAACCTGTGCCTCGCGTCTT	RT-PCR for Gdf3
		AGCGAGGCATGGAGAGAGCGGAGCAG	
63	Cripto	ATGGACGCAACTGTGAACATGATGTTCGCA	RT-PCR for Cripto
		CTTTGAGGTCCTGGTCCATCACGTGACCAT	
64	Dax1	TGCTGCGGTCCAGGCCATCAAGAG	RT-PCR for Dax1
		GGGCACTGTTCAGTTCAGCGGATC	
65	Esg1	GAAGTCTGGTTCCTTGGCAGGATG	RT-PCR for Esg1
		ACTCGATACACTGGCCTAGC	
66	Rex1	ACGAGTGGCAGTTTCTTCTTGGGA	RT-PCR for Rex1
		TATGACTCACTTCCAGGGGGCACT	
67	Nanog	AGGGTCTGCTACTGAGATGCTCTG	RT-PCR for Nanog
		CAACCACTGGTTTTTCTGCCACCG	
68	B-actin	GTGGGCCGCACAAGGCACCAA	RT-PCR for B-actin
		CTCTTTGATGTCACGCACGA	
69	Oct3/4	GGTTTTTTAGAGGATGGTTGAGTG	Methylation analysis of Oct3/4
		TCCAACCCTACTAACCCATCACC	
70	Nanog	GATTTTGTAGGTGGGATTAATTGTGAATTT	Methylation analysis of Nanog
		ACCAAAAAAACCCACACTCATATCAATATA	
71	GAPDH	TGTGTCCGTCGTGGATCTGA	Real time PCR primers and Probe
		CACCACCTTCTTGATGTCATCATAC	
		TGCCGCCTGGAGAAACCTGCC	
72	Oct3/4	ACCTTCAGGAGATATGCAAATCG	Real time PCR primers and Probe
		TTCTCAATGCTAGTTCGCTTTCTCT	
		AGACCCTGGTGCAGGCCCGG	
73	Nanog	GCAGAAAAACCAGTGGTTGAAGA	Real time PCR primers and Probe
		GCAATGGATGCTGGGATACTC	
		AGCAATGGTCTGATTCAGAAGGGCTCAG	
74	Wwp2	CAGCAGTGGCTTAGCCAATG	Real time PCR primers and Probe
		ACCGACGATTCTTCAGGTTCA	
		CACAGTGAATGAGGAACCTACTCCAGCCA	
75	Fbxw7	CCATGCAAAGTCTCAGATTATACCA	Real time PCR primers and Probe
		TCAGGTCCCCAAAAGTTGTTG	
		ACCACTGGCCTTGTACCATGTTCAGCA	
76	Wwp2	TTTAACTCGAGCTGAGGCTGCTGTCTCACAC	Wwp2 Amplification Primers
		AAGCCGCGGCCGCGGCTGCTGATTCTTTATTGC	
77	Wwp2	TCGGTATTGCCAGTTTCTTGTAGACTAAAGAATCAGCAGC	Wwp2 Mutagenesis Primers

Table S2. PCR primers for amplifying miRNA from DNA and RT-PCR/real time PCR primers

		GCTGCTGATTCTTTAGTCTACAAGAAACTGGCAATACCGA	
78	Fbxw7	TTTAACTCGAGAAAGCAGACATGATGAATTTTG	Fbxw7 Amplification Primers
		AAGCCGCGGCCGCTAACATGAAAAAACACATTTTAT	
79	Fbxw7	GACGACTCTCTAAATCCAACCAGGTGGAATTATTCTTTG	Fbxw7 Mutagenesis Primers 1
		CAAAGAATAATTCCACCTGGTTGGATTTAGAGAGTCGTC	
80	Fbxw7	CTATAACTTAAGTGGAATAAAATGTGTTTTTTCATGTTAGCGG	Fbxw7 Mutagenesis Primers 2
		CGCTAACATGAAAAAACACATTTTATTCCACTTAAGTTATAAG	
