# Supplementary material for: MiR-25 Regulates Wwp2 and Fbxw7 and Promotes Reprogramming of Mouse Fibroblast Cells to iPSCs
Source: PLoS One. 2012 Aug 17;7(8):e40938. doi: 10.1371/journal.pone.0040938 (PMC3422229; doi:10.1371/journal.pone.0040938)
Supplement: Table S3 — Conservation of miR-25 and miR-298 mature sequences. All mature sequences of miR-25 and miR-298 are obtained from miRBase database. (PDF) [file pone.0040938.s003.pdf]

Table S3. Conservation of miR-25 and miR-298 during evolution

Symbol	Species	Mature Sequence	Accession
hsa-miR-25	Homo sapiens	cauugcacuugucucggucuga	MIMAT0000081
ptr-mir-25	Pan troglodytes	cauugcacuugucucggcuga	MIMAT0002771
ggo-mir-25	Gorilla gorilla	cauugcacuugucucggucuga	MIMAT0002760
lla-mir-25	Lagothrix lagotricha	cauugcacuugucucggucuga	MIMAT0002779
mne-mir-25	Macaca nemestrina	cauugcacuugucucggucuga	MIMAT0002782
mml-mir-25	Macaca mulatta	cauugcacuugucucggucuga	MIMAT0002774
ppa-miR-25	Pan paniscus	cauugcacuugucucggucuga	MIMAT0002765
ppy-mir-25	Pongo pygmaeus	cauugcacuugucucggucuga	MIMAT0002768
bta-mir-25	Bos taurus	cauugcacuugucucggucuga	MIMAT0003853
cfa-mir-25	Canis familiaris	cauugcacuugucucggucuga	MIMAT0006697
rno-mir-25	Rattus norvegicus	cauugcacuugucucggucuga	MIMAT0000795
mmu-mir-25	Mus musculus	cauugcacuugucucggucuga	MIMAT0000652
mdo-mir-25	Monodelphis domestica	cauugcacuugucucggucuga	MIMAT0004179
dre-mir-25	Danio rerio	cauugcacuugucucggucuga	MIMAT0001793
fru-mir-25	Fugu rubripes	cauugcacuugucucggucuga	MIMAT0002914
tni-mir-25	Tetraodon nigroviridis	cauugcacuugucucggucuga	MIMAT0002915
hsa-mir-298	Homo sapiens	agcagaagcagggagguucuccca	MIMAT0004901
ptr-mir-298	Pan troglodytes	agcagaagcagggagguucuccca	MIMAT0008081
mml-mir-298	Macaca mulatta	agcagaagccgggugguucuccca	MIMAT0006254
rno-mir-298	Rattus norvegicus	ggcagaggagggcuguucuuccc	MIMAT0000900
mmu-mir-298	Mus musculus	ggcagaggagggcuguucuuccc	MIMAT0000376
