# Supplementary material for: MiR-25 Regulates Wwp2 and Fbxw7 and Promotes Reprogramming of Mouse Fibroblast Cells to iPSCs
Source: PLoS One. 2012 Aug 17;7(8):e40938. doi: 10.1371/journal.pone.0040938 (PMC3422229; doi:10.1371/journal.pone.0040938)
Supplement: Table S4 — Candidate target genes of miR-25. The table lists 54 genes based on microarray expression and Sylamer analysis following transfection of miR-25 mimic to the Dgcr8-deficient ES cells. (PDF) [file pone.0040938.s004.pdf]

Table S4. Candidate target genes of miR-25

Symbol	Description	Transf.FC	EntrezID
Rab8b	RAB8B, member RAS oncogene family	-2.543	235442
Whsc1l1	Wolf-Hirschhorn syndrome candidate 1-like 1 (human)	-2.395	234135
Tmem184b	transmembrane protein 184b	-2.389	223693
Ttc39b	tetratricopeptide repeat domain 39B	-2.325	69863
Nck2	non-catalytic region of tyrosine kinase adaptor protein 2	-2.052	17974
Lmbr1l	limb region 1 like	-1.986	74775
Fbxw7	F-box and WD-40 domain protein 7	-1.978	50754
Adam23	a disintegrin and metallopeptidase domain 23	-1.971	23792
Plekhm1	pleckstrin homology domain containing, family M (with RUN domain) member 1	-1.966	353047
Znrf2	zinc and ring finger 2	-1.925	387524
Acbd4	acyl-Coenzyme A binding domain containing 4	-1.907	67131
Wwp2	WW domain containing E3 ubiquitin protein ligase 2	-1.905	66894
Bag3	BCL2-associated athanogene 3	-1.899	29810
Cno	cappuccino	-1.89	117197
Spryd4	SPRY domain containing 4	-1.864	66701
Tle1	transducin-like enhancer of split 1, homolog of Drosophila E(spl)	-1.844	21885
Snx18	sorting nexin 18	-1.833	170625
Cdkn1c	cyclin-dependent kinase inhibitor 1C (P57)	-1.826	12577
Gnaq	guanine nucleotide binding protein, alpha q polypeptide	-1.82	14682
Gtf2e1	general transcription factor II E, polypeptide 1 (alpha subunit)	-1.794	74197
LOC100044124	similar to Nedd4 binding protein 2	-1.711	100044124
Pols	polymerase (DNA directed) sigma	-1.676	210106
Rnf4	ring finger protein 4	-1.671	19822
Efr3a	EFR3 homolog A (S. cerevisiae)	-1.652	76740
Pcolce2	procollagen C-endopeptidase enhancer 2	-1.643	76477
1810055G02Rik	RIKEN cDNA 1810055G02 gene	-1.63	72056
Pkdcc	protein kinase domain containing, cytoplasmic	-1.621	106522
Mtap	methylthioadenosine phosphorylase	-1.62	66902
Ppcs	phosphopantothenoylcysteine synthetase	-1.61	106564
Zfc3h1	zinc finger, C3H1-type containing	-1.603	216345
Eif4g2	eukaryotic translation initiation factor 4, gamma 2	-1.587	13690
Ccnc	cyclin C	-1.585	51813
Ibtk	inhibitor of Bruton agammaglobulinemia tyrosine kinase	-1.584	108837

Table S4. Candidate target genes of miR-25

Prkar1a	protein kinase, cAMP dependent regulatory, type I, alpha	-1.568	19084
Necap1	NECAP endocytosis associated 1	-1.546	67602
Hus1	Hus1 homolog (S. pombe)	-1.535	15574
Leprot	leptin receptor overlapping transcript	-1.526	230514
Dbt	dihydrolipoamide branched chain transacylase E2	-1.499	13171
Wrnip1	Werner helicase interacting protein 1	-1.487	78903
Osbpl2	oxysterol binding protein-like 2	-1.485	228983
Sfxn1	sideroflexin 1	-1.481	14057
Ric8b	resistance to inhibitors of cholinesterase 8 homolog B (C. elegans)	-1.48	237422
Rnf38	ring finger protein 38	-1.473	73469
Rhpn2	rhophilin, Rho GTPase binding protein 2	-1.444	52428
Fnip1	folliculin interacting protein 1	-1.423	216742
Snn	stannin	-1.418	20621
Bcat2	branched chain aminotransferase 2, mitochondrial	-1.406	12036
Brms1l	breast cancer metastasis-suppressor 1-like	-1.369	52592
Snap29	synaptosomal-associated protein 29	-1.356	67474
Nsmaf	neutral sphingomyelinase (N-SMase) activation associated factor	-1.35	18201
Adam19	a disintegrin and metallopeptidase domain 19 (meltrin beta)	-1.345	11492
Gcnt2	glucosaminyl (N-acetyl) transferase 2, I-branching enzyme	-1.343	14538
Dpy30	dpy-30 homolog (C. elegans)	-1.342	66310
Kat2b	K(lysine) acetyltransferase 2B	-1.304	18519
